# Homeostatic Regulation of Energetic Arousal During Acute Social Isolation: Evidence From the Lab and the Field

**DOI:** 10.1177/09567976231156413

**Published:** 2023-03-28

**Authors:** Ana Stijovic, Paul A. G. Forbes, Livia Tomova, Nadine Skoluda, Anja C. Feneberg, Giulio Piperno, Ekaterina Pronizius, Urs M. Nater, Claus Lamm, Giorgia Silani

**Affiliations:** 1 Department of Clinical and Health Psychology, Faculty of Psychology, University of Vienna; 2 Department of Cognition, Emotion, and Methods in Psychology, Faculty of Psychology, University of Vienna; 3 Department of Psychology, University of Cambridge; 4 Department of Psychology, Sapienza University of Rome; 5 University of Vienna Research Platform “The Stress of Life (SOLE) – Processes and Mechanisms Underlying Everyday Life Stress”

**Keywords:** social isolation, food deprivation, energetic arousal, fatigue, COVID-19, open data, preregistered

## Abstract

Recent evidence suggests that social contact is a basic need governed by a social homeostatic system. Little is known, however, about how conditions of altered social homeostasis affect human psychology and physiology. Here, we investigated the effects of 8 hr of social isolation on psychological and physiological variables and compared this with 8 hr of food deprivation in a lab experiment (*N* = 30 adult women). Social isolation led to lowered self-reported energetic arousal and heightened fatigue, comparable with food deprivation. To test whether these findings would extend to a real-life setting, we conducted a preregistered field study during a COVID-19 lockdown (*N* = 87 adults; 47 women). The drop in energetic arousal after social isolation observed in the lab replicated in the field study for participants who lived alone or reported high sociability, suggesting that lowered energy could be part of a homeostatic response to the lack of social contact.

Social contact is considered a basic need in many animals (Cacioppo et al., 2014), and, like our need for food, it may be governed by a dedicated regulatory system referred to as “social homeostasis” (Matthews & Tye, 2019). This idea is supported by studies in rodents and nonhuman primates showing that even relatively short periods of social isolation can have significant psychological and physiological effects both during development (Orben et al., 2020) and in adulthood (Tomova et al., 2021). Socially isolated rodents, for example, show behavioral signs of distress and depressive-like coping (Hilakivi et al., 1989; Takatsu-Coleman et al., 2013), increased secretion of stress hormones (e.g., corticosterone; Takatsu-Coleman et al., 2013), and a heightened tendency to seek social contact (Niesink & Van Ree, 1982). Comparable findings have been reported in nonhuman primates (see Løseth et al., 2014, for a review).

In humans, prolonged states of loneliness—the distress felt by the perception that one’s need to connect is not being met (Hawkley & Cacioppo, 2010)—has been shown to have detrimental health consequences (Bzdok & Dunbar, 2020). Yet only one study has investigated the effects of experimentally induced short-term social isolation (Tomova et al., 2020). Ten hours of social isolation resulted in lower happiness, higher discomfort, and an increased motivation to seek social contact. Using functional MRI, the researchers found that midbrain responses to social cues measured after isolation were comparable with responses to food cues after fasting. This suggests that craving for social contact and craving for food engage shared neural processes (Tomova et al., 2020). Here, we built on and extended this work by examining the extent to which the effects of experimentally induced isolation are comparable with the effects of food deprivation on psychological as well as physiological responses—specifically momentary stress, mood, and fatigue, measures commonly used as markers of psychological well-being (Doerr et al., 2021; Wilhelm & Schoebi, 2007), and physiological markers of stress responses, including salivary cortisol (sCort), salivary alpha-amylase (sAA), and heart rate (HR). To ensure the generalizability of our findings from the lab to a real-life setting, we collected momentary affective states multiple times per day during COVID-19 lockdown from an independent sample of participants undergoing an equivalent period of social isolation as those in the lab.

In the lab study, we induced social isolation in a within-subjects experimental setting and directly compared its effects with those of food deprivation. Participants came to the lab on 3 separate days and spent 8 hr each day in one of the experimental conditions: social isolation (no social contact but normal food intake), food deprivation (social contact but no food intake), and baseline (both social contact and normal food intake). Physiological and psychological measures were collected repeatedly across each session (see Fig. 1).

**Fig. 1.**
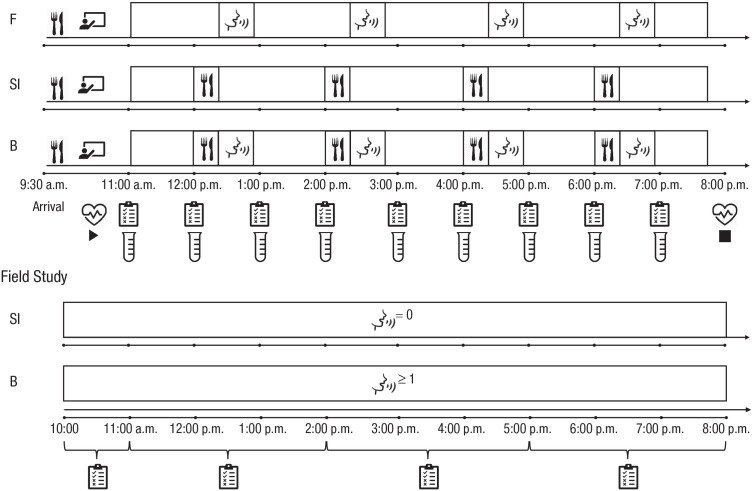
Overview of the procedure for the laboratory and field studies. Each session of the laboratory study (top) started at 11:00 a.m. and ended at 8:00 p.m. Before each session, participants received breakfast and training on how to complete the questionnaires and provide the saliva samples. In the baseline (B) condition, meals (snacks and a lunch) and social interaction slots (each 30-min long, filled with prescheduled video/audio calls with friends and family) happened every second hour. In the social isolation (SI) condition, meals were identical to those in the baseline condition, but there were no social interaction slots. In the food (F) deprivation condition, social interaction slots were identical to those in the baseline condition, but there were no meals. Questionnaires and saliva samples were collected hourly, and heart rate was collected continuously. Each data collection day in the field study (bottom) started between 10:00 a.m. and 11:00 a.m. and ended between 5:00 p.m. and 8:00 p.m. Questionnaires were collected at four random times within this period, with a minimum of 90 min between each prompt. The only days included in the analysis were those on which participants responded to all four consecutive prompts between 10:00 a.m. and 8:00 p.m. On baseline (B) days, participants reported at least one social interaction. On social isolation (SI) days, participants reported no social interactions.

In the field study, we examined the effects of social isolation during the COVID-19 lockdown on the same psychological variables collected in the lab study. Using an ecological momentary assessment (EMA) approach, we sent participants prompts to complete short questionnaires on their smartphones 5 times per day for 1 week. Psychological states reported during 8 hr with no social contact (social isolation days) were compared with those reported during days in which they had at least one social interaction during the same 8-hr period (baseline^1^ days; see Fig. 1). Three person-related characteristics (living condition [living alone vs. living with others], sociability, and chronic stress), which, in the pilot sample, modulated the effect of isolation on psychological states in daily life, were preregistered and used in the final analyses.

**Statement of Relevance**

Extended periods of social isolation pose a major threat to our physical and mental health, but the psychobiological mechanisms underlying these effects remain unclear. It has been proposed that social animals, such as humans, have a dedicated social homeostatic system that, comparable with the regulation of food intake, regulates our need for contact with others. In a tightly controlled lab experiment, we compared the effects of 8 hr of social isolation with 8 hr of food deprivation and found striking similarities in participants’ energy and fatigue across these two states. We validated the effect of social isolation on energy in a naturalistic setting during COVID-19 lockdown. Our results suggest that lowered energy and heightened fatigue are parts of a homeostatic response to a lack of social contact and that these changes could be a precursor to the more detrimental effects of long-term isolation.

We predicted that experimentally induced social isolation would lead to lower mood, heightened fatigue, and heightened physiological and psychological stress. Given the homeostatic nature of the systems regulating social isolation and food deprivation, we expected that the effects of social isolation would to some extent resemble the effects of food deprivation. Finally, we predicted that social isolation during the COVID-19 lockdown would lead to similar results as those observed in the lab setting (see https://osf.io/6ynt3/ for the preregistered analysis plan). We preregistered our hypothesis that sociability, living condition (alone vs. with others), and chronic stress would moderate the effects of social isolation; however, these hypotheses were not directional.

## Open Practices Statement

The design and analysis plan for the lab study were not preregistered. The analysis plan for the field study, together with the pilot analysis, was preregistered on OSF (https://osf.io/6ynt3/). The data, analysis code, and analysis outputs for both the lab and the field study have been made publicly accessible via OSF (https://osf.io/s8xk9/). Information on sample-size determination, data exclusion, all manipulations, and all measures are reported in the respective Method sections below and in the Supplemental Material available online.

## Lab Study

### Method

#### Participants

The sample consisted of 30 healthy adult female participants between 18 and 33 years old (*M* = 22.57, *SD* = 3.1) who took hormonal contraceptives and were not severely lonely or socially isolated in their everyday lives (see Section S1 in the Supplemental Material for more details on exclusion criteria and Table S1 in the Supplemental Material for more detailed information on sample composition). An optimal sample size of 28 participants was determined a priori using G*Power software (Faul et al., 2007) with 80% power to detect small to medium effects (Cohen’s *f* = 0.14) of food deprivation on self-reported measures of stress and mood (MacCormack & Lindquist, 2019) with .05 α error probability and correlation among repeated measures of .5. Data from an additional two participants were collected to account for potential dropouts.

Participants received €195 for completing three experimental sessions and an additional €15 if they provided additional measures the day after each session. The study was approved by the Ethics Committee of the University of Vienna, and participants signed informed consent before participation in the study.

#### Procedure

After completing the online screening, eligible participants were scheduled for three experimental sessions corresponding to three experimental conditions in a counterbalanced order: baseline, social isolation, and food deprivation. We achieved high comparability between the baseline, social isolation, and food deprivation conditions in terms of location, available activities, social interaction type, and food, thus controlling for the novelty and complexity of the environment. Each testing day consisted of a training and an experimental session and was highly similar in structure and setting (see Fig. 1). Participants were informed about the condition for the given day on arrival to the lab. During training, they were prepared to independently provide self-report and physiological measures. Experimental sessions lasted 8 hr each, and participants spent them alone in a spacious and comfortable lab room where they had access to nonsocial leisure activities (provided in the room and brought from home). In all sessions, any contact with experimenters and access to the Internet, smartphones, and social reading material (e.g., magazines with pictures of people) were not permitted. In the baseline condition, meals and video/audio calls with friends and family were prescheduled so that there was one meal and one 30-min conversation every 2 hr (4 times altogether). Access to meals and communication devices (study phone and laptop) was exclusively granted at predefined times and without direct contact with the experimenter to ensure high comparability between sessions. Participants received alarm-triggered instructions regarding the delivery of food and communication devices, instructions on delivery and storage of saliva samples, and momentary questionnaires via an electronic device (iPod Touch) with an EMA application (iDialogPad app, G. Mutz, Cologne, Germany). They received a standardized breakfast 1 hr before the start of each experimental session and were asked not to eat breakfast before coming to the lab. For a more detailed description of the experimental procedures, see Section S2 in the Supplemental Material.

#### Measures

Before coming to the lab for the first time, participants provided person-related measures via an online questionnaire on the SoSci Survey platform (https://www.soscisurvey.de/en/index). These included, among other measures, demographic variables (age and gender), a social isolation index (Grant et al., 2009), and the UCLA Loneliness Scale (Russell, 1996). See Section S1 in the Supplemental Material for a full list of person-related measures.

At the beginning of each hour (0, 1, . . . 8 hr), participants completed a separate visual analogue scale (VAS; 0–100) on momentary stress, anxiety, feelings of control, and avoidance, chosen from the items previously used to monitor subjective stress levels in parallel with physiological stress markers (von Dawans et al., 2011). Every second hour (0, 2, 4, 6, 8), measures of mood, fatigue, and desire for social contact and food were collected, in addition to the hourly measures. Participants responded to each manipulation check item—such as desire for social contact, loneliness, desire for food, and hunger—on a separate VAS (0–100); these items were comparable with the self-report measures used in the study of Tomova et al. (2020). Mood was assessed using the *Multidimensional Mood State Questionnaire*, which consists of three dimensions: mood valence, calmness, and energetic arousal (Steyer et al., 2003). Each dimension was represented by four bipolar adjectives on 5-point Likert scales, the sum of which was used as a score for that dimension (score range = 4–20). Items used to measure momentary fatigue were adapted from the Multidimensional Fatigue Inventory (MFI; Lin et al., 2009) on the basis of the factor loadings and face validity while also being adapted to apply to experience in the present moment (Doerr et al., 2021). This scale included five items that participants responded to on 5-point Likert scales representing five subscales of the MFI: general fatigue, mental fatigue, physical fatigue, reduced motivation, and reduced activity. Every fourth hour (0, 4, 8), a one-item VAS (0–100) on boredom was collected.

Every hour, right after completing the questionnaires, participants accumulated saliva in their mouth for 2 min and then transferred it via a straw into SaliCap vials (IBL, Hamburg, Germany). These vials were kept cool during the session in a box with cool packs. After the session, saliva was kept at −20 °C until analysis (see Section S3 in the Supplemental Material for more details on collection procedure and storage of the samples). HR (in beats per minute) was collected continuously throughout each session via a commercial HR sensor (H10 chest strap, Polar Electro, Kempele, Finland). See Section S4 in the Supplemental Material for a list of measures beyond the scope of the present article.

#### Analysis

We conducted linear mixed-effect models (LMEMs) to test the effect of each deprived condition (food deprivation and social isolation compared with baseline) on stress (including items on stress, anxiety, feelings of control, and desire to avoid the current situation), mood (including subscales of mood valence, calmness, and energetic arousal), fatigue (including subscales of general fatigue, physical fatigue, mental fatigue, reduced activity, and reduced motivation), loneliness, hunger, desire for social contact, and desire for food. To test whether the number of hours spent in each deprived state (social isolation, food deprivation) linearly affected the outcome measures, we tested interactions between condition and time spent in the experimental sessions for each measure. We built LMEMs in two steps (Aguinis et al., 2021), in which the first model was designed to test for the main effect of condition while controlling for time. The second model had an identical structure but also included the interaction between condition and time. For more details on the LMEM analysis, see Section S5 in the Supplemental Material.

Levels of sCort and sAA were extracted from the samples in the Biochemical Laboratory of the University of Vienna (see Section S3). For seven samples, sAA was below the detection threshold, so these were treated as missing data points. There were no missing values for sCort. Prior to the analysis, momentary sCort and sAA values were logarithmically transformed (*ln*(*x*) + 10; see Table S5 in the Supplemental Material for results with the raw data). Participants with outlier baseline values were removed, which resulted in the exclusion of one participant from analysis (*n* = 29 for sCort and sAA; see Section S6 in the Supplemental Material for details on outlier exclusion and Table S6 in the Supplemental Material for results on the full sample). Beats per minute were recorded for each second and were aggregated per minute before the analysis. Three participants were excluded from the HR analysis because of software bugs that resulted in deletion of recordings for the baseline condition (*n* = 27). The LMEMs used to test for the main effect of condition and the interaction between condition and time on momentary measures of sCort, sAA, and HR were identical to the ones used for the psychological measures. All *p* values from the analysis on physiological stress markers were Bonferroni corrected (see Section S6 for additional analyses, including area under the curve with respect to ground [AUCg] and with respect to increase [AUCi]).

To test the comparability of the effects of social isolation and food deprivation on stress, mood, and fatigue, we conducted a post hoc Bayesian repeated measures analysis of variance (ANOVA) in *JASP* (Version 0.16.3; JASP Team, 2022) for each outcome measure that was significantly altered in the same direction both by social isolation and food deprivation. Bayesian ANOVAs followed the structure of the respective LMEMs with two within-subjects factors of condition (baseline, social isolation, and food deprivation) and time (number of hours, here treated as a categorical variable). A post hoc contrast between social isolation and food deprivation was conducted to assess the probability for the absence of differences, which we would interpret as social isolation and food deprivation having comparable (i.e., indistinguishable) effects (see Section S5 in the Supplemental Material for more details).

Although the study was conducted in a tightly controlled environment, it is possible that the effects of social isolation were confounded by the effects of boredom. To test this, we ran a post hoc mediation analysis in which we aimed to determine whether the effect of isolation on fatigue and energy was mediated by boredom (see Section S7 in the Supplemental Material).

### Results

#### Short-term social isolation increased momentary loneliness and desire for social contact

**Fig. 2.**
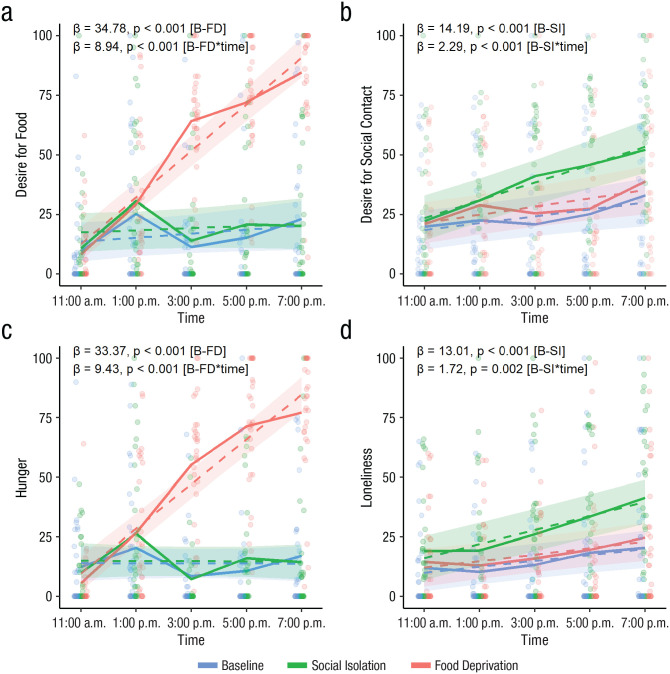
Momentary desire for food (a), desire for social contact (b), hunger (c), and loneliness (d) throughout the day in the lab study, separately for the baseline, social isolation, and food deprivation conditions. Dots represent raw data points. Solid lines indicate raw data, and dashed lines and ribbons indicate model estimates of the interaction effects between condition and time and their respective 95% confidence intervals. B = baseline, SI = social isolation, and FD = food deprivation.

Participants reported feeling lonelier, β = 13.01, *SE* = 3.42, *t*(29) = 3.8, *p* < .001, and having a higher desire for social contact, β = 14.19, *SE* = 3.8, *t*(29) = 3.74, *p* < .001, in the social isolation condition compared with baseline and feeling hungrier, β = 33.37, *SE* = 2.61, *t*(417) = 12.77, *p* < .001, and having higher desire for food, β = 34.78, *SE* = 2.69, *t*(417) = 12.91, *p* < .001, in the food deprivation condition compared with baseline. The effect sizes of food deprivation on hunger and desire for food were, however, almost 2 times higher than the effects of social isolation on loneliness and desire for social contact, respectively (see Table S4 in the Supplemental Material for relative effect sizes, i.e., standardized coefficients). The type of deprivation had specific effects on motivation, as participants reported neither more loneliness and desire for social contact in the food deprivation condition nor more hunger and desire for food in the social isolation condition (*p* > .25) compared with baseline. Interactions between condition and time showed that desire for social contact, β = 2.29, *SE* = 0.61, *t*(328) = 3.75, *p* < .001, and loneliness, β = 1.72, *SE* = 0.54, *t*(357) = 3.19, *p* = .002, increased with the number of hours in social isolation, and hunger, β = 9.43, *SE* = 0.76, *t*(415) = 12.49, *p* < .001, and desire for food, β = 8.94, *SE* = 0.69, *t*(328) = 13.06, *p* < .001, increased with the number of hours of food deprivation more steeply than in the baseline condition (see Fig. 2). Although loneliness and desire for social contact increased more steeply during isolation, simple-slopes analysis revealed that the increase in loneliness and desire for social contact was significant both during the isolation session—desire for social contact: β = 3.73, *SE* = 0.52, *t*(92) = 7.15, *p* < .001; loneliness: β = 2.96, *SE* = 0.38, *t*(357) = 7.75, *p* < .001—and during the baseline session—desire for social contact: β = 1.45, *SE* = 0.52, *t*(92) = 2.77, *p* = .007; loneliness: β = 1.24, *SE* = 0.38, *t*(357) = 3.26, *p* = .001. On the other hand, hunger and desire for food increased only during the food deprivation session—desire for food: β = 9.78, *SE* = 0.58, *t*(95.47) = 16.91, *p* < .001, hunger: β = 9.4, *SE* = 0.53, *t*(415) = 17.61, *p* < .001—and not during the baseline session (*p* > .15).

#### Short-term social isolation and food deprivation comparably modulated subjective energetic arousal, fatigue, and the desire to avoid the current situation

**Fig. 3.**
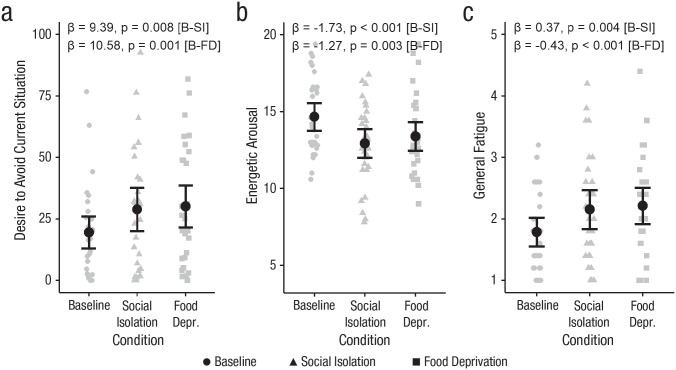
Momentary reports on psychological states in the lab study: desire to avoid the current situation (a), energetic arousal (b), and general fatigue (c) in the baseline, social isolation, and food deprivation conditions. Gray symbols represent raw data points. Black dots represent the main effect model estimates, and error bars represent 95% confidence intervals. B = baseline, SI = social isolation, and FD = food deprivation.

Participants reported a higher desire to avoid the current situation while they were isolated, β = 9.39, *SE* = 3.32, *t*(29) = 2.83, *p* = .008, and hungry, β = 10.58, *SE* = 2.92, *t*(29) = 3.61, *p* = .001, compared with baseline (see Fig. 3a). Comparability of these effects was supported by a large Bayes factor (BF_01_ *U* = 10.79), indicating strong evidence for the absence of a difference between the social isolation and the food deprivation condition. Furthermore, they reported lower energetic arousal, β = −1.73, *SE* = 0.42, *t*(29) = −4.11, *p* < .001, and higher general fatigue, β = 0.37, *SE* = 0.12, *t*(29) = 3.11, *p* = .004, during social isolation, as well as during food deprivation—energetic arousal: β = −1.27, *SE* = 0.4, *t*(29) = −3.21, *p* = .003; general fatigue: β = 0.43, *SE* = 0.12, *t*(29) = 3.67, *p* < .001 (see Figs. 3b and 3c). We found moderate evidence that the effects of social isolation and food deprivation on general fatigue (BF_01_ *U* = 9.13) and energetic arousal (BF_01_ *U* = 3.71) were comparable. Participants also reported reduced motivation and reduced activity both during isolation—reduced motivation: β = 0.27, *SE* = 0.13, *t*(29) = 2.08, *p* = .046; reduced activity: β = 0.39, *SE* = 0.14, *t*(29) = 2.71, *p* = .011—and during food deprivation—reduced motivation: β = 0.44, *SE* = 0.12, *t*(29) = 3.63, *p* = .001, reduced activity: β = 0.35, *SE* = 0.13, *t*(29) = 2.6, *p* = .015. We found strong evidence that the effects of social isolation and food deprivation on reduced activity (BF_01_ *U* = 10.17) were comparable, but for reduced motivation (BF_01_ *U* = 2.34) there was inconclusive evidence regarding the comparability of the effects across the two conditions. Neither of the deprived states modulated subjective levels of stress (*p* > .3) or anxiety (*p* > .27).

Finally, some mood changes occurred during food deprivation but not social isolation, including lower mood valence, β = −1.4, *SE* = 0.4, *t*(29) = −3.67, *p* < .001; calmness, β = −0.85, *SE* = 0.41, *t*(29) = −2.06, *p* = .049; and feelings of control, β = −6.62, *SE* = 2.57, *t*(29) = −2.58, *p* = .015, as well as higher mental fatigue, β = 0.28, *SE* = 0.1, *t*(29) = 2.69, *p* = .012. In addition, interactions between food deprivation and time showed that the desire to avoid the current situation, β = 1.8, *SE* = 0.47, *t*(688) = 3.83, *p* < .001, and general fatigue, β = 0.08, *SE* = 0.03, *t*(35.51) = 2.52, *p* = .017, increased and feelings of control, β = −1.33, *SE* = 0.65, *t*(29) = −2.06, *p* = .049; mood valence, β = −0.23, *SE* = 0.07, *t*(328) = −3.16, *p* = .002; and calmness, β = −0.17, *SE* = 0.07, *t*(328) = −2.03, *p* = .043, decreased with the number of hours spent in food deprivation relative to baseline. Our post hoc mediation analysis revealed that boredom only partially mediated the effect of isolation on energy and fatigue (see Section S7 in the Supplemental Material for details). See Tables S3 and S7 in the Supplemental Material for results from the additional measures beyond the scope of the present article.

#### Short-term social isolation did not modulate physiological markers of stress

**Fig. 4.**
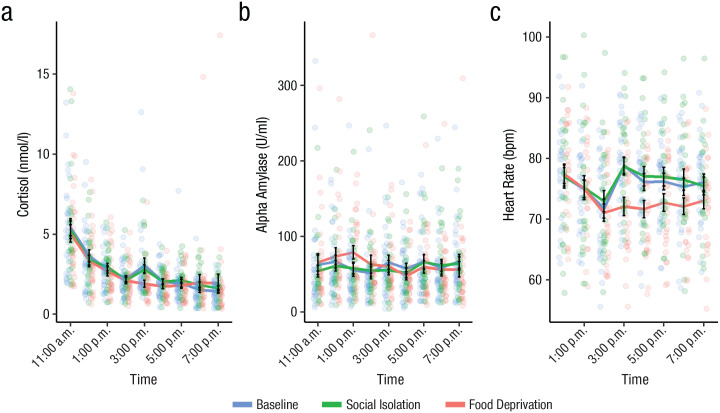
Salivary cortisol (a), salivary alpha-amylase (b), and heart rate (c) throughout the day in the lab study, separately for the baseline, social isolation, and food deprivation conditions. Raw momentary values for salivary measures for nine time points and beats per minute (BPM) measures are aggregated per hour. Colored dots represent raw data points, black dots represent mean values for each time point, and error bars represent standard errors around the respective means.

Momentary sCort (nmol/l), sAA (U/ml), and HR (beats per minute) were analyzed with LMEMs (see Fig. 4 for raw momentary values). We tested whether being in a deprived state led to an increase in the physiological markers of stress and whether there was an increase with the duration spent in deprivation relative to baseline.

Momentary levels of sCort and sAA were not modulated by either social isolation or food deprivation after *p* values had been Bonferroni corrected (*p* > .12). HR was lower in the food deprivation than in the baseline condition, β = −2.64, *SE* = 0.65, *t*(26) = −4.04, *p* < .001, whereas it was not modulated by the social isolation condition (*p* = 1). Furthermore, HR decreased more steeply during the day in the food deprivation condition compared with baseline, β = −0.56, *SE* = 0.15, *t*(26) = −3.66, *p* = .001. Momentary sAA decreased during the day spent in food deprivation more steeply than in the baseline session, β = −0.02, *SE* = 0.01, *t*(665) = −3.66, *p* < .001, but this was not the case in social isolation (*p* = 1). Simple-slopes analysis showed that sAA decreased during the day only in the food deprivation session, β = −0.02, *SE* = 0.01, *t*(109) = −3.01, *p* = .003, and not in the baseline session (*p* = .14). Interactions between deprived conditions and time were also not significant for sCort (*p* = 1). See Tables S2 and S4 in the Supplemental Material for a more detailed overview of the results from the lab study.

## Field Study

### Method

#### Participants

Eighty-seven participants (47 female, 54.02%; age: *M* = 32.95 years, *SD* = 12.95, range = 18–70) were included in the final sample. They were in Austria, Italy, or Germany during the COVID-19 lockdown between April and May 2020; 62 participants completed the study in German, and 25 completed it in Italian. Thirty participants lived alone at the time of testing (35%), whereas 57 lived with at least one other person (see Section S8 and Table S1 in the Supplemental Material for more detailed sample characteristics). On average, participants had 3.02 (*SD* = 1.46) days classified as baseline days and 1.46 (*SD* = 0.76) days classified as social isolation days (see the Subsample Selection Procedure section).

Participants received €20 on completion of the study and were entered into a draw to win an additional €100 voucher. The study was approved by the Ethics Committee of the University of Vienna, and participants provided informed consent before starting the study.

#### Procedure and measures

We used data collected as part of a larger EMA project investigating the effects of the COVID-19 lockdown on behavior and well-being (Feneberg et al., 2022; for further explanation, see the preregistration at https://osf.io/6ynt3/). Data were collected during two lockdowns: in April and May 2020 (Burst 1) and in November and December 2020 (Burst 2). For 7 consecutive days, participants were prompted to complete a short survey on a smartphone app (movisens, Karlsruhe, Germany) between the following time points: 10:00 a.m. to 11:00 a.m., 11:00 a.m. to 2:00 p.m., 2:00 p.m. to 5:00 p.m., and 5:00 p.m. to 8:00 p.m. In addition to the four app-initiated prompts, the last data entry of the day was self-initiated in the evening just before participants went to sleep (data from this prompt were not included in the present study). All prompts were time stamped, and during each entry, participants reported the behaviors they had engaged in since the last prompt or were currently engaged in and their momentary states. The behaviors included the number of social interactions^2^ since the last prompt (“Have you had an uninterrupted social exchange of more than 2 minutes since the last entry/since getting up?” to which participants could answer yes or no and specify a number) and the activity they were currently engaged in (“What were you doing just before this assessment?” to which participants could select one of the following: working, studying, free time, or other). The momentary states—including loneliness (“At the moment, I feel lonely”), stress (“In the moment, I feel stressed”), fatigue (“In the moment, I feel exhausted”), and desire for social contact (“How strong is your momentary desire for social exchange?”)—were each assessed on a separate VAS (from 0, *not at all*, to 100, *very*). In addition to the single item measures, participants also completed a multidimensional mood questionnaire consisting of three subscales: mood valence, calmness, energetic arousal (Wilhelm & Schoebi, 2007), assessed by the same type of VAS.

After downloading the app, participants provided their age, gender, and the number of people in their household (i.e., living condition). After the 7-day period, they completed a series of questionnaires, including the UCLA Loneliness Scale (Russell, 1996), the Social Reward Questionnaire (SRQ; Foulkes et al., 2014), and the Perceived Stress Scale (Klein et al., 2016; Mondo et al., 2021).

#### Subsample selection procedure

To simulate the conditions in the lab study (social isolation vs. baseline day) in the field study, we analyzed days during the EMA period in which participants responded to the first four prompts (i.e., those between 10:00 a.m. and 8:00 p.m.), and we created two levels of the variable “type of day.” On social isolation days, participants reported having no social interaction in any of the first four EMA prompts of the day. On baseline days, participants reported having at least one social interaction in at least one of the first four EMA prompts of the day. To determine the proportion of participants who had at least one social isolation day and one baseline day during the study period and could therefore be included in the analysis, we conducted a pilot analysis using the data from the second lockdown (*N* = 357) as part of our preregistered analysis plan. Sensitivity analysis showed that expected sample size (~11.5% of the full sample, i.e., 109 participants) would provide us with 80% power for determining medium effect sizes (Cohen’s *d* = 0.6) for the main effects and small effect sizes (Cohen’s *d* = 0.2) for interactions. As outlined above, the final sample consisted of 9.14% of the 951 participants (*n* = 87), which was slightly below this estimate.

#### Analysis

All analyses of the field study were preregistered (see https://osf.io/6ynt3/). To compare the differences in momentary loneliness, desire for social contact, stress, fatigue, and the three mood measures on social isolation days versus baseline days, we conducted an LMEM for each of these dependent variables. In each model, we included the following variables: type of day, which indicated whether this was a baseline day (0) or a social isolation day (1); free time, a dummy-coded variable that indicated whether participants were engaged in free time at the time of the data entry (0) or whether they were working or studying (i.e., not free time, 1); and EMA time, the time of the assessment centered on participants’ first assessment of the day. This was included in all models to control for diurnal changes in stress and mood (Feneberg et al., 2022). Next, to determine the influence on the number of hours spent in isolation on loneliness, desire for social contact, stress, fatigue, and the three mood measures, we included the interaction between type of day and EMA time in the above models.

In addition to the two models that addressed the same questions as the lab study, we decided to explore whether interindividual differences modulated the expected effects. The sample from the lab study was less diverse than the sample from the field study in terms of gender, age, living condition, loneliness, and chronic stress (see Section S1 in the Supplemental Material for the lab study inclusion criteria), which could explain different findings across studies. On the basis of the pilot analysis, we concluded that sociability, living alone, and chronic stress might have modulated the effects of social isolation. Thus, three additional models were included in the analysis. To determine whether living condition (dummy coded: alone = 0 vs. with others = 1), chronic stress, and sociability modulated the impact of type of day on momentary states (i.e., loneliness, desire for social contact, stress, fatigue, and mood), we ran the above models again but included the cross-level interaction between type of day and these person-related predictors. Finally, we conducted a post hoc analysis of the main effect of gender on study outcomes, as well as the gender interaction with type of day. See Section S9 in the Supplemental Material for further details on the LMEM analysis for the field study.

### Results

#### Momentary loneliness was higher but desire for social contact was lower during social isolation days compared with baseline

**Fig. 5.**
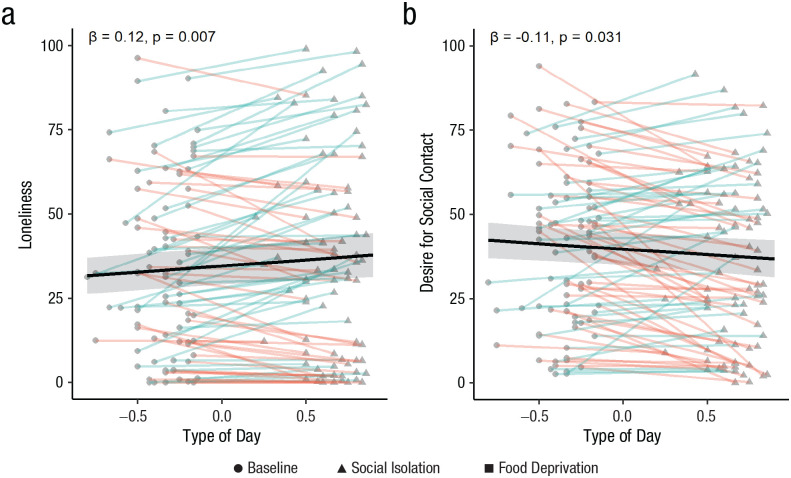
Momentary reports of loneliness (a) and desire for social contact (b) as a function of type of day in the field study, separately for the baseline and social isolation days (there is no food deprivation in the field study and the variable is called type of day, not condition). Given that the number of social isolation and baseline days varied between participants in the field study, type of day is represented as a continuous variable (baseline is < 0 and social isolation > 0). Dots represent raw data points. Red lines indicate a lower value on social isolation days, and blue lines indicate a higher value on social isolation days, compared with baseline. Black lines represent main effect model estimates, and ribbons represent 95% confidence intervals.

In line with our preregistered hypotheses, results showed that participants were lonelier, β = 3.64, *SE* = 1.31, *t*(87.24) = 2.77, *p* = .007 (see Fig. 5a), and showed a trend toward lower mood valence, β = −2.17, *SE* = 1.18, *t*(79.38) = −1.85, *p* = .068, on social isolation days compared with baseline. Contrary to our hypothesis, results showed that participants desired less social contact, β = −3.22, *SE* = 1.47, *t*(74.79) = −2.20, *p* = .031, on social isolation days compared with baseline (see Fig. 5b). There was no significant difference in stress, calmness, energetic arousal, or fatigue on social isolation days compared with baseline (*p* > .15). Time of day did not interact with type of day (i.e., social isolation vs. baseline) for any of the above momentary state measures (*p* > .08).

**Fig. 6.**
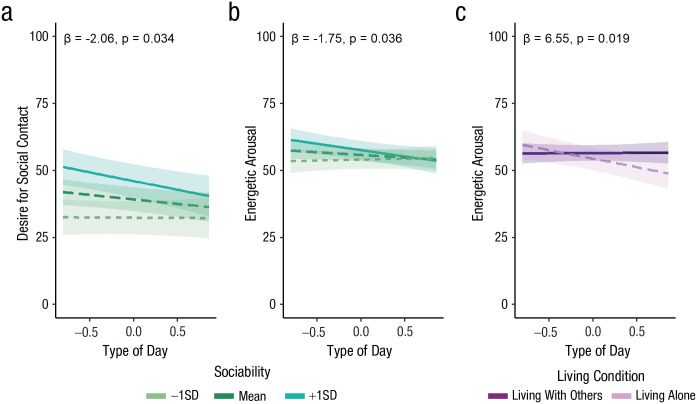
Interactions between person-related characteristics and type of day in the field study (model estimates). The graphs show the interaction between type of day and sociability for desire for social contact (a), the interaction between type of day and sociability for energetic arousal (b), and the interaction between type of day and living condition for energetic arousal (c) as a function of type of day. Given that the number of social isolation and baseline days varied between participants in the field study, type of day is represented as a continuous variable (baseline is < 0 and social isolation > 0). Lines represent model estimates of the interaction effects between type of day and person-related characteristics. Ribbons indicate 95% confidence intervals.

A lower desire for social contact on social isolation days was particularly prominent in more sociable individuals. There was a significant interaction between sociability and type of day for momentary desire for social contact, β = −2.06, *SE* = 0.95, *t*(66.65) = −2.17, *p* = .034 (see Fig. 6a). Participants scoring higher on trait sociability (+1 *SD*) had significantly lower desire for social contact on social isolation days compared with baseline days, β = −6.52, *SE* = 2.12, *t*(66.65) = −3.07, *p* = .003, but this was not the case for those scoring lower (−1 *SD*) on sociability, β = −0.18, *SE* = 2.08, *t*(66.65) = −0.09, *p* = .929. There was a trend toward a significant interaction between living condition and type of day for momentary loneliness, β = −5.52, *SE* = 2.82, *t*(80.69) = −1.96, *p* = .054. People living alone showed significantly higher loneliness on social isolation days compared with baseline days, β = 6.94, *SE* = 2.32, *t*(80.69) = 2.99, *p* = .004, but this was not the case for those living with others, β = 1.42, *SE* = 1.61, *t*(80.69) = 0.88, *p* = .380. Chronic stress did not modulate the effect of social isolation on any dependent variable (*p* > .1).

#### Energetic arousal was lower on social isolation days, compared with baseline, in more sociable individuals and in individuals who lived alone

Sociability interacted with type of day for energetic arousal, β = −1.75, *SE* = 0.82, *t*(69.16) = −2.14, *p* = .036 (see Fig. 6b). Simple-slopes analysis revealed that participants scoring higher on trait sociability (+1 *SD*) reported lower energetic arousal on social isolation days compared with baseline days, β = −4.69, *SE* = 1.84, *t*(69.16) = −2.55, *p* = .013, but this was not the case for those scoring lower (−1 *SD*) on sociability, β = 0.70, *SE* = 1.79, *t*(69.16) = 0.39, *p* = .696. Living condition (alone vs. with others) also interacted with type of day for energetic arousal, β = 6.55, *SE* = 2.72, *t*(68.27) = 2.41, *p* = .019 (see Fig. 6c). Participants living alone had significantly lower energetic arousal on social isolation days compared with baseline, β = −6.41, *SE* = 2.25, *t*(68.27) = −2.84, *p* = .006, but this was not the case for those living with others, β = 0.15, *SE* = 1.58, *t*(68.27) = 0.09, *p* = .927. See Tables S4 and S8 in the Supplemental Material for a more detailed overview of the field study results. Gender was not related to any of the outcomes and also did not significantly interact with type of day (*p* > .110; see Table S9 in the Supplemental Material).

## Discussion

We investigated the effects of 8 hr of acute social isolation both in a tightly controlled lab experiment and in a field study during COVID-19 lockdown. In the lab, we compared the effect of social isolation with the effect of food deprivation and found greater fatigue and lower energetic arousal in both deprived states, relative to a baseline day, when participants had both food and social interactions. In the field, we found evidence suggesting that our results from the lab may generalize to a real-world setting—acute social isolation during lockdown was related to lower energetic arousal in more sociable participants and those who lived alone.

In the lab, we observed that food deprivation and social isolation led to similar changes in energetic arousal and fatigue, a striking finding given that food deprivation involves a direct deprivation of energy in terms of calories, whereas social isolation does not. Lowered energy could be part of a homeostatic response to a lack of social contact (Matthews & Tye, 2019) and could result from hypervigilance that drains energy resources (Quadt et al., 2020). Quadt et al. proposed that social isolation makes people expand more energy when trying to cope with stressors because they overpredict the energy demands of the environment, resulting in metabolic, cardiovascular, and immune dysregulation (Hawkley & Cacioppo, 2010; Quadt et al., 2020). Moreover, regulation of social behavior and energy are deeply linked in many social species (IJzerman et al., 2015; Koto et al., 2015). For example, it has been shown that being with conspecifics reduces the energy costs spent by other homeostatic systems, such as for nutritional regulation (Koto et al., 2015) and thermoregulation, possibly via increasing the predictability of the environment (IJzerman et al., 2015). Although social isolation is more detrimental over prolonged periods (Quadt et al., 2020), our findings demonstrate that changes in subjective energetic arousal and fatigue also occur even after a relatively short period of social isolation.

Changes in energy and fatigue following acute social isolation could be a precursor to the more detrimental effects of long-term isolation. Loneliness shows a strong bidirectional relationship with feelings of energy (Hawkley et al., 2010; Holding et al., 2020) and momentary perceived social isolation is related to higher sleepiness, fatigue, and lower energy the following day (Hawkley et al., 2010). Feeling sleepier and more fatigued is related to a lower desire to initiate social interactions (Holding et al., 2020) and higher momentary loneliness (Hawkley et al., 2010). This bidirectional link between social isolation and energy results in a feedback loop in which social isolation and loneliness reinforce each other. Therefore, investigating the effects of acute social isolation could elucidate the precursors and potential mechanisms of the long-term effects of loneliness and social isolation, which could prove useful for interventions. It is nevertheless also important to note that the acute effects of isolation may be qualitatively different from chronic effects rather than just being different in magnitude or severity (Saporta et al., 2021).

In the field, we observed that acute social isolation during lockdown was related to lower energetic arousal in more sociable participants and those who lived alone. These analyses were preregistered without hypotheses about the direction of these effects. However, the dependence of social isolation effects on social context and personality aligns with the model of social homeostasis, as the set points of the system do not work on a unified scale but are flexible and depend on the subjective nature of social experiences (Matthews & Tye, 2019). Indeed, several studies have demonstrated that *perceived* social support is better at predicting outcomes to stressful events than *received* social support (Prati & Pietrantoni, 2010), especially during the COVID-19 pandemic (Grey et al., 2020). The validation of our laboratory findings in a more diverse sample and in context of daily life is especially valuable, given the unique context of the COVID-19 lockdown and that the laboratory study was conducted on young participants with low levels of loneliness and objective social isolation.

We found that energetic arousal of participants who lived alone during the lockdown was most affected by isolation compared with those who lived with others. In everyday life, having the potential to interact with someone, especially in person, may be an important factor for mitigating the effects of social isolation. Thus, the reassurance that someone is there if we need them or knowing that we can interact with someone later may help to mitigate the effects of short-term social isolation in everyday life. Finally, we found that although sociable participants had lower energy when isolated compared with days on which they had social interaction, this effect was absent in less sociable participants. This finding is consistent with results of a previous study conducted during the COVID-19 pandemic, in which people who reported being more socially active before the pandemic were more affected by the reduction in their social contacts in terms of their depressive symptoms (Sommerlad et al., 2022).

Interestingly, the observed changes in energetic arousal and fatigue in the lab were not accompanied by changes in physiological and subjective stress or other measures of mood. The hypothalamic-pituitary-adrenal axis is consistently activated by situations that are perceived as uncontrollable (Dickerson & Kemeny, 2004); however, participants did not report changes in their feelings of control during social isolation or increased stress or anxiety. Thus, although participants may have found social isolation uncomfortable, as demonstrated by their increased desire to avoid the situation, they knew when their isolation would end, which may have increased perceived control over the situation. This interpretation is consistent with findings from space psychology showing that having a precise end date can protect against the negative effects of isolation (Riva et al., 2022). Furthermore, participants’ loneliness in the field study was greater on social isolation days; however, their desire for social contact was reduced compared with days on which they had an interaction. Although contrary to our predictions, this fits with studies of loneliness that show it is linked to reduced social approach behavior, possibly because lonely individuals interpret their social environment as more threatening (Cacioppo & Cacioppo, 2018).

The study has some limitations that need to be considered. Although our experimental design enabled us to isolate effects of social isolation and food deprivation with high precision, this may have come at the cost of ecological validity. More robust changes in psychological states following isolation may occur if, during the baseline condition, participants engaged in social interactions in person rather than remotely and could freely use their smartphones or if the social isolation period were longer. It is possible that additional factors associated with isolation, such as boredom, played a role in influencing energy and fatigue. Our post hoc mediation analysis revealed that boredom only partially mediated the effect of isolation on energy and fatigue. This highlights that future studies will need to carefully disentangle boredom effects from those of isolation. Because our lab sample consisted only of women, the findings cannot be generalized to men. In the field study, however, our exploratory analysis showed that gender was not related to any of the outcomes, and it did not interact with type of day. Given recent evidence suggesting gender-related neurophysiological effects of chronic loneliness (Morr et al., 2022; Spreng et al., 2020), future studies will need to test the effects of acute social isolation on energy and fatigue in men as well as women to confirm the generalizability of these findings. It is worth highlighting the correlational nature of the field study so we could not establish the causality of the found effects. It is possible that more sociable participants decided to refrain from social interactions on days in which they felt low in energy rather than a lack of interactions resulting in low energy. Although these possibilities are not mutually exclusive, our lab study provides evidence for the latter interpretation. Future experience-sampling studies could use an event-contingent recording approach to help elucidate the direction of these effects in the field. Finally, in the field study, some participants may have chosen to be alone, and periods of solitude can have positive affective consequences when actively chosen (Nguyen et al., 2018).

To conclude, we compared the effects of short-term social isolation in a tightly controlled lab experiment and in a field study during COVID-19 lockdown. Across these two different contexts, we found consistent effects of social isolation on people’s energy, which in the lab study were comparable with the effects of food deprivation. This suggests that lowered energy could be part of a homeostatic response to a lack of social contact. Understanding the short-term effects of social isolation could prove useful in elucidating the precursors and potential mechanisms of the long-term effects of social isolation.

## Supplemental Material

sj-docx-1-pss-10.1177_09567976231156413 – Supplemental material for Homeostatic Regulation of Energetic Arousal During Acute Social Isolation: Evidence From the Lab and the Field Supplemental material, sj-docx-1-pss-10.1177_09567976231156413 for Homeostatic Regulation of Energetic Arousal During Acute Social Isolation: Evidence From the Lab and the Field by Ana Stijovic, Paul A. G. Forbes, Livia Tomova, Nadine Skoluda, Anja C. Feneberg, Giulio Piperno, Ekaterina Pronizius, Urs M. Nater, Claus Lamm and Giorgia Silani in Psychological Science

## Transparency

*Action Editor:* Daniela Schiller

*Editor:* Patricia J. Bauer

*Author Contributions*

Ana Stijovic and Paul A. G. Forbes share first authorship of this article.

**Ana Stijovic:** Conceptualization; Formal analysis; Investigation; Methodology; Project administration; Visualization; Writing – original draft; Writing – review & editing.

**Paul A. G. Forbes:** Conceptualization; Formal analysis; Investigation; Methodology; Project administration; Writing – original draft; Writing – review & editing.

**Livia Tomova:** Methodology; Writing – review & editing.

**Nadine Skoluda:** Data curation; Methodology; Writing – review & editing.

**Anja C. Feneberg:** Investigation; Project administration; Writing – review & editing.

**Giulio Piperno:** Investigation; Project administration; Writing – review & editing.

**Ekaterina Pronizius:** Investigation; Project administration; Writing – review & editing.

**Urs M. Nater:** Conceptualization; Data curation; Funding acquisition; Methodology; Resources; Writing – review & editing.

**Claus Lamm:** Conceptualization; Funding acquisition; Methodology; Resources; Supervision; Writing – review & editing.

**Giorgia Silani:** Conceptualization; Funding acquisition; Methodology; Resources; Supervision; Writing – review & editing.

## Notes

1. In the experimental study, baseline was an experimental session in which participants had both food and social contact; in the field study, baseline was an 8-hr period in which participants had social interaction. The same term was used for the sake of comparability of the lab and the field study.

2. A social interaction was defined as a continuous social exchange (which could be face to face or via audio, video, or text) between the participant and at least one other person that lasted at least 2 min. The 2-min criterion was chosen to ensure that participants thought of the number of back-and-forth exchanges with others rather than short one-way exchanges such as single emails, texts, or fleeting exchanges (e.g., a short greeting to a neighbor in the hallway or on the street). All participants were informed that such short one-way interactions did not count as social interactions.
