# Supplementary material for: Homeostatic Regulation of Energetic Arousal During Acute Social Isolation: Evidence From the Lab and the Field
Source: Psychol Sci. 2023 Mar 28;34(5):537–51. doi: 10.1177/09567976231156413 (PMC13029414; doi:10.1177/09567976231156413)
Supplement: sj-docx-1-pss-10.1177_09567976231156413 – Supplemental material for Homeostatic Regulation of Energetic Arousal During Acute Social Isolation: Evidence From the Lab and the Field [file sj-docx-1-pss-10.1177_09567976231156413.docx]

**Supplemental Materials for the article**

“Homeostatic regulation of energetic arousal during acute social isolation-evidence from the lab and the field”

[Laboratory study 2](#_Toc117783171)

[S1. Inclusion criteria 2](#_Toc117783172)

[S2. Experimental procedure for the laboratory study 3](#_Toc117783173)

[S3. Collection and analysis of salivary measures 5](#_Toc117783174)

[S4. Additional measures 6](#_Toc117783175)

[S5. General information about linear mixed models in the laboratory study 7](#_Toc117783176)

[S6. Additional information on the physiological data analysis 8](#_Toc117783177)

[S7. Post-hoc mediation analyses on boredom 9](#_Toc117783178)

[Table S1. Demographic characteristics of the lab study and the field study samples 10](#_Toc117783179)

[Table S2. Means, standard deviations, and confidence intervals of dependent variables for the lab study 11](#_Toc117783180)

[Table S3. Means, standard deviations, and confidence intervals of dependent variables for the morning after each session of the lab study 15](#_Toc117783181)

[Table S4. Overview of the results from the experimental and the field study. 16](#_Toc117783182)

[Table S5. Results of momentary salivary cortisol and alpha amylase on the full sample before outlier exclusion, with transformed data (log10). 18](#_Toc117783183)

[Table S6. Results of momentary salivary cortisol and alpha amylase on the full sample before outlier exclusion, with raw data 18](#_Toc117783184)

[Table S7. Overview of the results from the morning after each experimental session. 19](#_Toc117783185)

[Field study 20](#_Toc117783186)

[S8. Comparison of the field sample to population norms 20](#_Toc117783187)

[S9. General information about linear mixed models in the field study 20](#_Toc117783188)

[Table S8. Between-person means, standard deviations, and confidence intervals of dependent variables for the field study 21](#_Toc117783189)

[Table S9. Post-hoc analyses – effects of gender in the field study 22](#_Toc117783190)

[References 23](#_Toc117783191)

# Laboratory study

S1. Inclusion criteria

Inclusion criteria were: female sex, German language proficiency (mother tongue or at least C1 level), a body mass index (BMI) between 18 and 25, identifying as heterosexual, and taking hormonal contraceptives. Salivary cortisol (sCort) and alpha amylase (sAA) vary depending on sex and accounting for sex-differences would have required a much larger sample size, given the complexity of the study (Strahler et al., 2017). Since the phase of the menstrual cycle has been shown to modulate affiliative motivation (Maner & Miller, 2014), only women who took hormonal contraceptives, and thus had no monthly variation in the sex hormones progesterone and estrogen, were selected.

Additionally, participants were excluded if they had any current or previous neurological or psychiatric disorders, or a history of drug or alcohol abuse, if they smoked more than 5 cigarettes a day, if they used medications known to affect endocrine functioning aside from the hormonal contraceptives, and if they were pregnant or nursing. To exclude the potential effects of long-term states of isolation and loneliness, participants were excluded if their score on the UCLA Loneliness Scale was over two standard deviations from the normative mean of the student population (Russell, 1996), and if they had high objective social isolation as measured by a social isolation index (Grant et al., 2009). A high index of social isolation was based on Grant et al., i.e., responding to two or three of the following questions with a “yes”: “I live alone”, “I see my friends less than once a month”, “I see my family less than once a month”. Finally, participants were excluded if their score on a chronic stress scale (a screening scale [SSCS] from the Trier Inventory of Chronic Stress) (Schulz et al., 2004) was at least two standard deviations from the normative mean and if their global score on the Eating Disorder Questionnaire (EDE-Q)(Hilbert et al., 2007) was above a clinical threshold (Rø et al., 2015).

S2. Experimental procedure for the laboratory study

Participants were recruited via email advertisements delivered through the electronic subject recruitment system of the Faculty of Psychology of University of Vienna (SONA systems). After completing the online screening, eligible participants were contacted by the experimenters to schedule the three experimental sessions (baseline, social isolation, and food deprivation) in a counterbalanced order. The gap between the sessions was, on average, 9.85 days (SD = 3.4, min = 5, max = 22). Testing was not performed during the menstrual period, to avoid potential confounding effects of menstrual pain and discomfort. Each session lasted around 11 hours and consisted of: training (~2h), a test period (8h), and the experimental tasks (1h). Participants arrived in the morning after fasting overnight and they all received the same breakfast upon arrival (three pieces of toast with marmalade and a glass of juice, 360 kCal). They were then informed which session they would complete on that day. During the training, participants learned how to provide saliva samples using the passive drool method, how to use the application on the iPod Touch (iDialogPad App, G. Mutz, Cologne) (see Linnemann et al., 2017), and about the structure of the session, such as when and how they would eat and have social interaction during the day. Participants were explicitly informed that sleeping and exercise were not allowed and were made aware of the presence of a web camera in the room, which projected in the experimenter’s room. The web camera was used only by experimenter during the session to check on the well-being of participants and to ensure compliance with the experimental schedule. No video recordings of participant were made.

Participants spent the test period alone in a spacious room equipped with chairs, tables, and the measurement equipment (see Figure S1). Non-social free time activities were available in the test room in all conditions, such as sudoku, puzzles, and books with non-social contents (e.g., books about plants or technology). Participants were also instructed to bring 3-5 activities from home after checking their eligibility with the experimenter before the session. Social stimuli, including novels, books, or magazines with images of faces, any devices with access to internet (e.g., phones, laptops, tablets), were not allowed in order to control social engagement across all sessions. In the baseline condition, participants had access to both food and social contact. Meals were delivered every two hours, including three small snacks (at +1, +5, and +7h; banana, butter cookies, crackers respectively, each 120 kCal) and one lunch (at +3h; spaghetti with bolognese/pesto sauce, 600 kCal). Four 30-min social interaction sessions were scheduled right after the meals and were done via audio or video calls with friends and family (scheduled by participants prior to each testing day). We chose to restrict social contact to that with friends and family, rather than the experimenter, as it has been argued that close social connections are crucial for fulfilling our need for contact (Cacioppo et al., 2014) and that only interactions with close people regulate our mood (Quoidbach et al., 2019). However, because it was not feasible to enable visits of participants’ friends and family to the lab, social interaction took place remotely. Food and the communication device (e.g., phone or laptop) were left by experimenter in front of the test room at predefined times, which was done to avoid direct contact between the participant and the experimenter. The phone and laptop used for social interactions were not the personal items of the participants, were only available during the social interaction slots, and had no access to any contents other than phone numbers (phone) and Skype application (laptop). This was done to avoid the social interaction slots being used as smartphone/internet breaks instead of actual period of social interaction. Participants were instructed to make maximal use of the conversation slots and meals. The amount of consumed food, the time spent in conversation, and medium used for conversation were recorded. 85% of meals were eaten, 8.6% were not eaten, and in 6.1% of cases data was missing. In an average conversation slot, participants used 93% of given time for conversation (28 out of 30min). For 70% of the conversation slots participants used a phone for audio calls and in 15% they used video calls with Skype (in 15.8% of slots this data was missing).

Every hour started with a measurement point prompted by an alarm. Each measurement point started with participants filling out questionnaires which took between 1-5 minutes to complete. After that, participants provided a saliva sample following the instructions on the iPod and placed it in a cooling box.


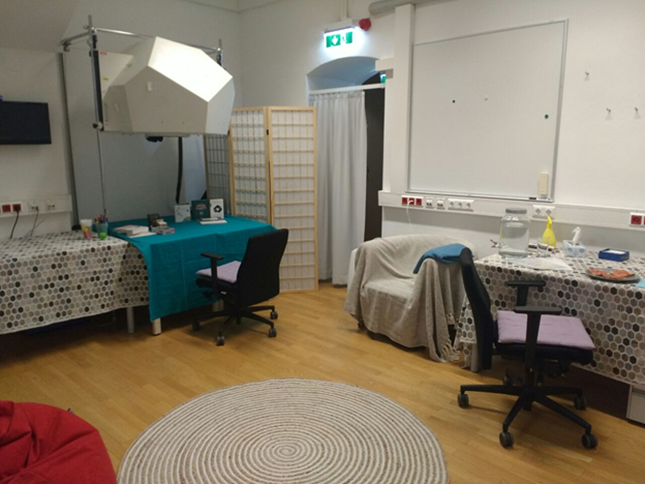
Data collection took part between November 2019 and November 2020, with a pause between March and May 2020 due to the first COVID-19 lockdown in Austria. No differences were found in any of the psychological or physiological measures between participants who took part in the experiment before and after the lockdown, therefore, analyses were performed without controlling for this factor.

**SM Figure 1**. A preview of the room in which experimental sessions took place.

S3. Collection and analysis of salivary measures

Saliva was collected using the SaliCap system (IBL, Hamburg, Germany). Participants were instructed to accumulate saliva in their mouth for two minutes and transfer it via the passive drooling method into SaliCap vials. During the session, vials were kept among ice blocks. At the end of each session, they were stored at -20^○^C until the analysis. Saliva samples collected at home were kept in the participant’s freezer or fridge until the appointment with the experimenter. If samples were kept in the fridge, the appointment was not more than five days after the saliva collection.

Salivary cortisol and alpha amylase were used as markers of the two stress response systems, the hypothalamic-pituitary-adrenal (HPA) axis and the autonomic nervous system (ANS) respectively. Biochemical analyses were conducted in Biochemical Laboratory of the University of Vienna. Free cortisol concentrations in the saliva were measured using commercial luminescence immunosorbent assay (LUM [luminescence immunoassay]; IBL International, a Tecan Group, Hamburg, Germany). Salivary alpha-amylase activity was measured using a kinetic colorimetric test and reagents obtained from DiaSys Diagnostic Systems (Holzheim, Germany). Saliva was diluted 1:400 using a 0.9% saline solution. The reagents contained the enzyme alpha-amylase in a specified amount and alpha glucosidase, which converts the substrate ethyliden nitrophenyl to p-nitrophenol. The rate of formation of p-nitrophenol is directly proportional to the samples’ amylase activity and was detected using an absorbance reader at 405nm (Biotek Synergy HTX, BioTek Instruments, Winooski, VA, USA). Intra-und Inter-Assay coefficient of variation were < 10% for both alpha amylase and cortisol. Lower detection limit was 0.011 µg/dL (according to IBL-Tecan manual) for cortisol and 3 U/L for alpha amylase.

S4. Additional measures

Additional momentary mood measures were collected during the session (Profile of Mood States (Dalbert, 1992), and desire for phone and internet~~, and boredom~~ [VAS 1-100]). These measures are not reported herein and can be found in the data file for the laboratory study at the OSF (<https://osf.io/s8xk9/>). At the end of each session, participants completed experimental tasks beyond the scope of the present paper.

The morning after each session, participants were instructed to deliver two more saliva samples and respond to two more questionnaires (pen and paper); right upon awakening and 30min later. The first morning questionnaire was a questionnaire measuring sleep quality (adapted from (Buysse et al., 1989; Klaus et al., 2019). Results from this measure are not reported herein and can be found in the data file for the laboratory study at the OSF (<https://osf.io/s8xk9/>). The second morning questionnaire included the same momentary measures that were collected during the experiment, including momentary stress (items: stress, anxiety, feelings of control, desire to avoid present situation; (von Dawans et al., 2011), mood (MDBF; (Steyer et al., 2003), fatigue (items adapted for momentary assessment from the MFI; (Doerr et al., 2021; Lin et al., 2009), VAS (1-100) for desire for social contact and food, loneliness, and hunger. Cortisol awakening response (CAR) and alpha-amylase awakening response (AAR) were calculated by subtracting the value of the saliva sample collected upon awakening (s1) from the value of the saliva sample collected 30min later. Psychological momentary measures, CAR and AAR were analyzed on 28 participants, due to two participants missing and one providing invalid baseline measures. Like morning psychological measures, this analysis was performed on 28 participants, since one participant did not provide baseline morning measures and one provided invalid baseline measures due to late collection. Measures recorded the morning after each session were tested with LMMs that included the fixed effect of condition and a random intercept of each participant. We did not observe any effects on the day after either deprivation session compared to the day after the baseline session (*p* > .08). Results from this analysis are reported in the Table S3.

S5. General information about ~~linear mixed models in~~ the laboratory study analyses

In each linear mixed model, condition was used as the categorical predictor with the baseline condition as a reference category, meaning that each model tested contrasts between the baseline condition and each deprived state (i.e., baseline vs. isolation and baseline vs. food deprivation). Time was a continuous predictor, indicating the number of hours since the start of the session. It was centered at 11am - the time of the first measurement point. A separate model was conducted for each dependent measure. The structure of random effects was always kept “maximal” (Barr et al., 2013), which included random slopes for condition and time in the main effect models and, additionally, a random slope for interaction between condition and time in the interaction models. If models did not converge, complexity of the random effects was incrementally decreased. The “bobyqa” optimizer was used to improve the performance of the LMMs and thus decrease the chances that models would not converge (Powell, 2009). All models used restricted maximum likelihood estimation. Significance was tested using Satterthwaite's method with the R package *lmerTest* (Kuznetsova et al., 2017). Simple slopes analysis for significant interactions was performed with R packages *reghelper* (Hughes, 2021) and *interactions* (Long, 2019).

In the Bayesian ANOVA, we used the default multivariate Cauchy prior in JASP (*r* scale prior width for fixed effects of 0.5) and we tested against a custom null model that included the effect of time without condition. Bayes factors (*BF01*) between 3 and 10 were considered moderate and those larger than 10 were considered strong evidence for the null hypothesis (van Doorn et al., 2021). JASP outputs and the data for this analysis are available online (<https://osf.io/s8xk9/>).

S6. Additional information on the physiological data analysis

After transformation of the momentary values of sCort and sAA (*ln(x)* + 10), outlier values were identified from the first measurements (before the start) of each session, which would indicate that participants already started a condition with higher sCort or sAA values. The threshold for exclusion was more than three standard deviations higher or lower than the average baseline value for a given condition (Stalder et al., 2016).

Exploratory analyses were performed on physiological markers of stress. Area under curve with respect to ground (AUCg) and area under curve with respect to increase (AUCi) (Pruessner et al., 2003) were calculated using raw momentary values of sCort, sAA, and HR. LMMs with the fixed effect of condition and a random intercept of each participant were used to test the effects of condition on the AUC measures. All p-values from the analysis of the physiological stress markers were Bonferroni corrected for three comparisons per measure (LMM on momentary values, AUCg, and AUCi). Results from the analyses are reported together with the main results in the Table S1.

S7. Post-hoc mediation analyses on boredom


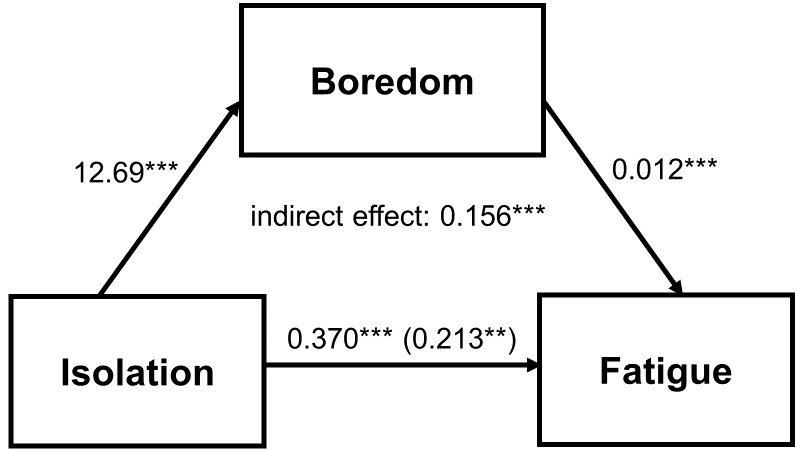

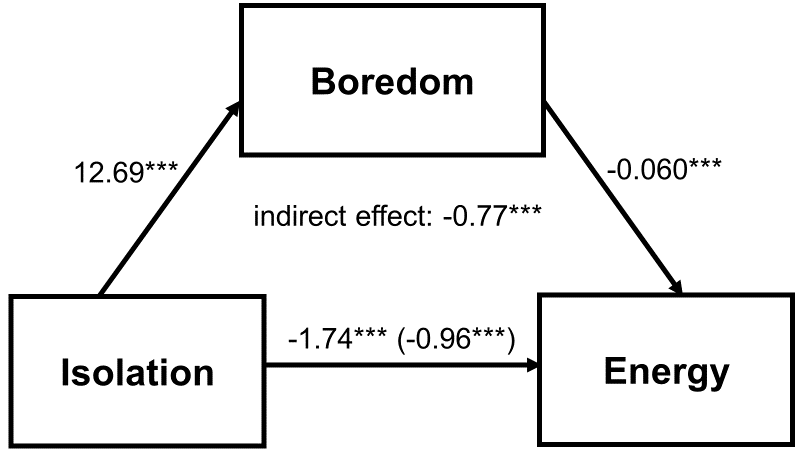


To determine whether the effects of isolation on energy and fatigue in the lab study were mediated by boredom, we ran a post-hoc mediation analysis using the R package *mediation* (Tingley et al., 2014). In all models, we included time as a fixed effect and included a random intercept for participant and a random slope for time (all code is available online <https://osf.io/s8xk9/>). We found a significant indirect effect of isolation on fatigue via boredom (β = 0.16, 95% *CI* [0.09, 0.24], *p* < .001), but this did not fully account for the direct effect (β = 0.21, 95% *CI* [0.06, 0.37], *p* = .010). Similarly, we found a significant indirect effect of isolation on energy via boredom (β = -0.77, 95% *CI* [-1.11, -0.47], *p* < .001), but this did not fully account for the direct effect (β = -0.96, 95% *CI* [-1.48, -0.47], *p* < .001). Thus, the analyses show that boredom partially, but not fully, mediated the effect of isolation on fatigue and energy. The figure shows the unstandardized coefficients for each mediation model with direct effects shown in brackets (*** *p* < .001; ** *p* < .01).

Table S1. Demographic characteristics of the lab study and the field study samples

| Person-related characteristics | Lab study | Field study |
| --- | --- | --- |
| Gender | 30 female (100%) | 47 female (54%), 40 male (46%) |
| Age | *M* = 22.47 (*SD* = 3.16) | *M* = 32.95 (*SD* = 12.95) |
| Living condition | 28 do not live alone (93.3 %), 2 live alone (6.7%) | 57 do not live alone (65.5%), 30 live alone (34.5%) |
| Loneliness (UCLA scale; Russel, 1996) | *M* = 32.43 (*SD* = 6.43) | *M* = 41.57 (*SD* = 9.87) |
| Sociability (SRQ; Foulkes et al., 2014) | - | *M* = 4.96 (*SD* = 1.61) |
| Chronic stress (PSS; Klein et al., 2016; Mondo et al., 2021) | - | *M* = 19.15 (*SD* = 7.48) |

Table S2. Means, standard deviations, and confidence intervals of dependent variables for the lab study

| Condition | Dependent variable | |  | | | | | Measurement point | | | | |
| --- | --- | --- | --- | --- | --- | --- | --- | --- | --- | --- | --- | --- |
|  |  |  | Overall | 11:00 | 12:00 | 13:00 | 14:00 | 15:00 | 16:00 | 17:00 | 18:00 | 19:00 |
|  |  |  | *M* (*SD*) 95% *CI* | | | | | | | | | |
| Baseline | Motivation [1-100] | loneliness | 14.87 (22.65) [6.77, 22.98] | 12.03 (19.17) [5.17, 18.89] | - | 10.3 (16.4) [4.43, 16.17] | - | 13.27 (21.04) [5.74, 20.79] | - | 18.37 (27.52) [8.52, 28.22] | - | 20.4 (29.1) [9.99, 30.81] |
|  |  | desire for soc. contact | 24.31 (27.26) [14.56, 34.07] | 19.87 (23.85) [11.33, 28.4] | - | 22.57 (24.17) [13.92, 31.22] | - | 20.93 (27.99) [10.92, 30.95] | - | 25.2 (30.06) [14.44, 35.96] | - | 33 (30.22) [22.19, 43.81] |
|  |  | hunger | 13.83 (22.15) [5.9, 21.75] | 12.43 (19.88) [5.32, 19.55] | - | 20.43 (25.45) [11.33, 29.54] | - | 8.53 (17.4) [2.31, 14.76] | - | 10.73 (22.39) [2.72, 18.75] | - | 17 (25.61) [7.83, 26.17] |
|  |  | desire for food | 16.94 (26.33) [7.52, 26.36] | 9.67 (18.82) [2.93, 16.4] | - | 25.3 (30.57) [14.36, 36.24] | - | 11.33 (20.62) [3.96, 18.71] | - | 15.27 (28.73) [4.99, 25.55] | - | 23.13 (32.92) [11.35, 34.91] |
|  | Stress  [1-100] | stress | 11.61 (18.28) [5.07, 18.15] | 14.03 (19.34) [7.11, 20.95] | 10.43 (14.98) [5.07, 15.79] | 10.37 (15.51) [4.82, 15.92] | 9.33 (16.67) [3.37, 15.3] | 9.23 (15) [3.87, 14.6] | 12 (19.27) [5.1, 18.9] | 11.37 (19.16) [4.51, 18.22] | 13.27 (21.99) [5.4, 21.13] | 14.43 (22.63) [6.34, 22.53] |
|  |  | anxiety | 5.35 (11.83) [1.12, 9.59] | 7.03 (13.85) [2.08, 11.99] | 5.63 (12.71) [1.08, 10.18] | 4.3 (8.69) [1.19, 7.41] | 5.1 (10.2) [1.45, 8.75] | 4.63 (8.64) [1.54, 7.73] | 4.8 (8.36) [1.81, 7.79] | 4.63 (11.75) [0.43, 8.84] | 5.33 (15.18) [-0.1, 10.77] | 6.7 (17.09) [0.58, 12.82] |
|  |  | control. | 79.45 (21.31) [71.83, 87.08] | 77.57 (23.91) [69.01, 86.12] | 83.97 (18.66) [77.29, 90.64] | 81.2 (19.74) [74.14, 88.26] | 80.33 (21.27) [72.72, 87.95] | 80.37 (18.98) [73.58, 87.16] | 77.3 (20.97) [69.79, 84.81] | 76.7 (24.68) [67.87, 85.53] | 78.77 (21.34) [71.13, 86.4] | 78.87 (22.26) [70.9, 86.83] |
|  |  | avoidance | 19.48 (22.6) [11.39, 27.57] | 10.73 (18.94) [3.96, 17.51] | 8.97 (15.46) [3.43, 14.5] | 8.97 (15.19) [3.53, 14.4] | 14.13 (17.67) [7.81, 20.46] | 13.7 (21.58) [5.98, 21.42] | 19.33 (23.08) [11.07, 27.59] | 27.27 (29.79) [16.61, 37.93] | 31.83 (29.88) [21.14, 42.52] | 40.4 (31.83) [29.01, 51.79] |
|  | Mood  [4-20] | mood valence | 16.85 (2.7) [15.89, 17.82] | 16.8 (2.48) [15.91, 17.69] | - | 17.23 (2.53) [16.33, 18.14] | - | 17.6 (2.25) [16.79, 18.41] | - | 16.3 (2.91) [15.26, 17.34] | - | 16.33 (3.34) [15.14, 17.53] |
|  |  | calmness | 16.01 (2.98) [14.95, 17.08] | 16.07 (3.19) [14.92, 17.21] | - | 16.2 (2.47) [15.32, 17.08] | - | 16.67 (3.13) [15.55, 17.79] | - | 15.9 (2.35) [15.06, 16.74] | - | 15.23 (3.75) [13.89, 16.57] |
|  |  | energetic arousal | 14.66 (3.34) [13.46, 15.86] | 14.77 (3.59) [13.48, 16.05] | - | 15.53 (2.78) [14.54, 16.53] | - | 15.37 (2.94) [14.31, 16.42] | - | 14 (3.76) [12.65, 15.35] | - | 13.63 (3.64) [12.33, 14.94] |
|  | Fatigue  [1-5] | general fatigue | 1.79 (0.86) [1.48, 2.1] | 1.8 (0.96) [1.46, 2.14] | - | 1.53 (0.68) [1.29, 1.78] | - | 1.57 (0.77) [1.29, 1.84] | - | 1.93 (0.94) [1.6, 2.27] | - | 2.1 (0.96) [1.76, 2.44] |
|  |  | physical fatigue | 2.76 (1) [2.4, 3.12] | 2.57 (1.1) [2.17, 2.96] | - | 2.7 (1.15) [2.29, 3.11] | - | 2.6 (1) [2.24, 2.96] | - | 2.93 (0.91) [2.61, 3.26] | - | 3 (0.83) [2.7, 3.3] |
|  |  | mental fatigue | 2.39 (0.97) [2.05, 2.74] | 2.2 (0.92) [1.87, 2.53] | - | 2.03 (0.76) [1.76, 2.31] | - | 2.17 (1.02) [1.8, 2.53] | - | 2.67 (0.96) [2.32, 3.01] | - | 2.9 (1.18) [2.48, 3.32] |
|  |  | reduced motivation | 1.86 (0.96) [1.52, 2.2] | 1.8 (0.92) [1.47, 2.13] | - | 1.5 (0.63) [1.27, 1.73] | - | 1.87 (0.97) [1.52, 2.21] | - | 2.07 (1.17) [1.65, 2.49] | - | 2.07 (1.11) [1.67, 2.46] |
|  |  | reduced activity | 2.91 (1.14) [2.5, 3.31] | 3.07 (1.31) [2.6, 3.54] | - | 2.6 (1.19) [2.17, 3.03] | - | 2.67 (1.03) [2.3, 3.03] | - | 3.13 (1.04) [2.76, 3.51] | - | 3.07 (1.11) [2.67, 3.46] |
|  | Physiolog-ical stress responses | sAA in U/mL | 60.12 (48.52) [42.76, 77.49] | 60.66 (68.96) [35.98, 85.34] | 65.23 (46.27) [48.67, 81.78] | 54.21 (45.99) [37.75, 70.67] | 51.99 (42) [36.96, 67.02] | 63.96 (50.88) [45.75, 82.17] | 57.61 (38.38) [43.88, 71.35] | 64.77 (49.3) [47.13, 82.41] | 56.94 (45.68) [40.6, 73.29] | 65.75 (49.24) [48.13, 83.37] |
|  |  | sCort in nmol/L | 2.64 (1.49) [2.11, 3.17] | 5.31 (2.87) [4.29, 6.34] | 3.57 (2.04) [2.84, 4.3] | 2.8 (1.22) [2.37, 3.24] | 2.18 (1.01) [1.82, 2.54] | 3.04 (2.44) [2.17, 3.91] | 1.99 (0.99) [1.63, 2.34] | 1.97 (1.32) [1.5, 2.44] | 1.5 (0.85) [1.19, 1.8] | 1.42 (0.67) [1.18, 1.66] |
|  |  | heart rate (bpm)^1^ | 75.81 (9.64) [75.34, 76.28] | - | 77 (10.07) [76.51, 77.5] | 74.99 (9.99) [74.5, 75.47] | 71.77 (10.07) [71.28, 72.26] | 78.89 (9.36) [78.43, 79.34] | 76.14 (9.23) [75.69, 76.59] | 76.24 (9.42) [75.78, 76.7] | 75.35 (9.26) [74.9, 75.81] | 76.1 (9.7) [75.63, 76.57] |
| Isolation | Motivation [1-100] | loneliness | 27.89 (27.57) [18.02, 37.75] | 19.07 (21.78) [11.27, 26.86] | - | 19.27 (25.68) [10.08, 28.45] | - | 26.07 (27.25) [16.32, 35.82] | - | 33.57 (30.83) [22.54, 44.6] | - | 41.47 (32.29) [29.91, 53.02] |
|  |  | desire for soc. contact | 38.5 (29.43) [27.97, 49.03] | 22.37 (27.19) [12.64, 32.1] | - | 30.83 (27.53) [20.98, 40.68] | - | 41.2 (31) [30.11, 52.29] | - | 45.97 (31.19) [34.81, 57.13] | - | 52.13 (30.26) [41.3, 62.96] |
|  |  | hunger | 14.75 (21.49) [7.06, 22.44] | 9.83 (13.76) [4.91, 14.76] | - | 26.3 (30.49) [15.39, 37.21] | - | 7.17 (14.95) [1.82, 12.52] | - | 16.1 (22.72) [7.97, 24.23] | - | 14.33 (25.53) [5.2, 23.47] |
|  |  | desire for food | 19.3 (26.82) [9.7, 28.9] | 10.73 (20.25) [3.49, 17.98] | - | 30.67 (31.35) [19.45, 41.89] | - | 14.03 (24.74) [5.18, 22.89] | - | 20.83 (29.33) [10.34, 31.33] | - | 20.23 (28.45) [10.05, 30.41] |
|  | Stress [1-100] | stress | 11.43 (16.45) [5.55, 17.32] | 9.8 (15.76) [4.16, 15.44] | 6.63 (10.38) [2.92, 10.35] | 11.73 (16.19) [5.94, 17.53] | 11.8 (17.42) [5.56, 18.04] | 11.1 (15.68) [5.49, 16.71] | 11.27 (16.7) [5.29, 17.24] | 12.83 (17.35) [6.62, 19.04] | 15.2 (20.86) [7.73, 22.67] | 12.53 (17.69) [6.2, 18.86] |
|  |  | anxiety | 6.26 (11.9) [2, 10.52] | 5.97 (13.01) [1.31, 10.62] | 4.17 (8.05) [1.29, 7.05] | 6.57 (14.95) [1.22, 11.92] | 5.67 (13.04) [1, 10.33] | 6.57 (11.38) [2.49, 10.64] | 5.5 (9.16) [2.22, 8.78] | 10.37 (15.06) [4.98, 15.76] | 6.07 (10.28) [2.39, 9.74] | 5.43 (12.2) [1.07, 9.8] |
|  |  | controllability | 76.79 (24.98) [67.85, 85.73] | 79.27 (28.25) [69.16, 89.38] | 77.87 (26.88) [68.25, 87.49] | 79.4 (24.54) [70.62, 88.18] | 76.83 (23.99) [68.25, 85.42] | 74.73 (23.97) [66.16, 83.31] | 77.6 (22.49) [69.55, 85.65] | 74.97 (26.72) [65.4, 84.53] | 74.77 (25.15) [65.77, 83.77] | 75.7 (22.83) [67.53, 83.87] |
|  |  | avoidance | 28.87 (29.1) [18.46, 39.28] | 12.73 (19.31) [5.82, 19.64] | 18.37 (23.42) [9.98, 26.75] | 19.7 (24.4) [10.97, 28.43] | 21.9 (24.25) [13.22, 30.58] | 26.5 (28.94) [16.14, 36.86] | 27.5 (33.54) [15.5, 39.5] | 37.07 (35.58) [24.33, 49.8] | 46.13 (36.36) [33.12, 59.14] | 49.93 (36.1) [37.02, 62.85] |
|  | Mood [4-20] | mood valence | 16.25 (3.03) [15.17, 17.34] | 16.83 (2.68) [15.87, 17.79] | - | 16.7 (3) [15.63, 17.77] | - | 16.4 (3.32) [15.21, 17.59] | - | 16 (3.03) [14.92, 17.08] | - | 15.33 (3.12) [14.22, 16.45] |
|  |  | calmness | 16.1 (2.68) [15.14, 17.06] | 16.8 (2.25) [15.99, 17.61] | - | 16.4 (2.59) [15.47, 17.33] | - | 16.73 (2.43) [15.86, 17.6] | - | 15.8 (2.43) [14.93, 16.67] | - | 14.77 (3.71) [13.44, 16.09] |
|  |  | energetic arousal | 12.93 (3.55) [11.66, 14.2] | 14.03 (3.81) [12.67, 15.4] | - | 13.43 (3.51) [12.18, 14.69] | - | 12.7 (3.71) [11.37, 14.03] | - | 12.37 (3.22) [11.21, 13.52] | - | 12.13 (3.52) [10.87, 13.39] |
|  | Fatigue [1-5] | general fatigue | 2.15 (1.1) [1.76, 2.55] | 1.87 (1.04) [1.49, 2.24] | - | 1.87 (0.97) [1.52, 2.21] | - | 2.2 (1.24) [1.76, 2.64] | - | 2.2 (1.03) [1.83, 2.57] | - | 2.63 (1.19) [2.21, 3.06] |
|  |  | physical fatigue | 2.99 (1.11) [2.6, 3.39] | 2.63 (1.27) [2.18, 3.09] | - | 2.77 (1.17) [2.35, 3.18] | - | 3.07 (1.08) [2.68, 3.45] | - | 3.13 (1.01) [2.77, 3.49] | - | 3.37 (1.03) [3, 3.74] |
|  |  | mental fatigue | 2.58 (1.15) [2.17, 2.99] | 2.23 (1.14) [1.83, 2.64] | - | 2.27 (1.14) [1.86, 2.68] | - | 2.57 (1.14) [2.16, 2.97] | - | 2.7 (1.18) [2.28, 3.12] | - | 3.13 (1.14) [2.73, 3.54] |
|  |  | reduced motivation | 2.13 (1.2) [1.7, 2.56] | 1.8 (1.1) [1.41, 2.19] | - | 1.83 (0.99) [1.48, 2.19] | - | 2.1 (1.18) [1.68, 2.52] | - | 2.33 (1.32) [1.86, 2.81] | - | 2.6 (1.4) [2.1, 3.1] |
|  |  | reduced activity | 3.29 (1.09) [2.9, 3.68] | 2.97 (1.1) [2.57, 3.36] | - | 3.1 (1.06) [2.72, 3.48] | - | 3.43 (1.04) [3.06, 3.81] | - | 3.4 (1.1) [3.01, 3.79] | - | 3.57 (1.14) [3.16, 3.97] |
|  | Physiolog-ical stress responses | sAA in U/mL | 57.42 (42.01) [42.39, 72.46] | 52.49 (41.53) [37.63, 67.36] | 59.22 (38.54) [45.43, 73.01] | 56.34 (42.51) [41.13, 71.55] | 53.23 (44.43) [37.33, 69.13] | 54.16 (34.86) [41.68, 66.63] | 50.64 (34.78) [38.19, 63.08] | 66.6 (53.06) [47.62, 85.59] | 59.76 (42.76) [44.46, 75.06] | 64.37 (45.62) [48.04, 80.69] |
|  |  | sCort in nmol/L | 2.62 (1.34) [2.14, 3.1] | 5.11 (3.02) [4.03, 6.2] | 3.28 (1.73) [2.66, 3.9] | 2.87 (1.27) [2.42, 3.33] | 2.08 (0.99) [1.72, 2.43] | 2.77 (1.5) [2.23, 3.3] | 1.99 (0.86) [1.68, 2.3] | 2.1 (0.91) [1.77, 2.43] | 1.8 (0.98) [1.45, 2.15] | 1.58 (0.82) [1.29, 1.87] |
|  |  | heart rate (bpm)^1^ | 76.26 (10.49) [75.75, 76.77] | - | 77.02 (11.48) [76.45, 77.58] | 75.2 (11.81) [74.63, 75.78] | 72.99 (10.76) [72.47, 73.51] | 78.73 (9.39) [78.27, 79.19] | 77.1 (9.98) [76.61, 77.58] | 77.03 (9.6) [76.56, 77.5] | 76.54 (10.81) [76.02, 77.07] | 75.48 (10.12) [74.98, 75.97] |
| Fasting | Motivation [1-100] | loneliness | 17.53 (22.73) [9.4, 25.67] | 14.47 (20.11) [7.27, 21.66] | - | 12.97 (18.22) [6.45, 19.49] | - | 15.97 (18.93) [9.19, 22.74] | - | 19.63 (26.98) [9.98, 29.29] | - | 24.63 (29.43) [14.1, 35.17] |
|  |  | desire for soc. contact | 28.36 (27.08) [18.67, 38.05] | 21 (24.46) [12.25, 29.75] | - | 28.93 (25.27) [19.89, 37.98] | - | 25.5 (24.82) [16.62, 34.38] | - | 27.5 (28.1) [17.45, 37.55] | - | 38.87 (32.75) [27.15, 50.58] |
|  |  | hunger | 47.19 (23.83) [38.66, 55.72] | 5.5 (12.68) [0.96, 10.04] | - | 26.67 (26.92) [17.03, 36.3] | - | 55.27 (26.38) [45.83, 64.71] | - | 71.33 (27.24) [61.59, 81.08] | - | 77.2 (25.94) [67.92, 86.48] |
|  |  | desire for food | 51.72 (22.68) [43.6, 59.84] | 8.2 (14.59) [2.98, 13.42] | - | 29.37 (27.73) [19.45, 39.29] | - | 64.27 (21.25) [56.66, 71.87] | - | 72.13 (26) [62.83, 81.44] | - | 84.63 (23.84) [76.1, 93.16] |
|  | Stress [1-100] | stress | 13.63 (18.72) [6.93, 20.33] | 16.27 (20.65) [8.88, 23.65] | 10.63 (17.05) [4.53, 16.74] | 11.73 (18.19) [5.22, 18.24] | 13.27 (18.49) [6.65, 19.88] | 12.4 (17.38) [6.18, 18.62] | 9.5 (12.49) [5.03, 13.97] | 15.5 (20.47) [8.17, 22.83] | 15.73 (20.25) [8.49, 22.98] | 17.63 (23.52) [9.22, 26.05] |
|  |  | anxiety | 5.99 (11.36) [1.92, 10.05] | 9.3 (18.46) [2.69, 15.91] | 4.43 (7) [1.93, 6.94] | 5.1 (11.41) [1.02, 9.18] | 4.97 (8.53) [1.92, 8.02] | 5.3 (9.72) [1.82, 8.78] | 5.63 (8.92) [2.44, 8.82] | 5.07 (13.42) [0.26, 9.87] | 6.03 (11.48) [1.92, 10.14] | 8.03 (13.29) [3.28, 12.79] |
|  |  | controllability | 72.84 (24.16) [64.19, 81.48] | 76.7 (24.98) [67.76, 85.64] | 78.03 (19.34) [71.11, 84.96] | 75.9 (22.14) [67.98, 83.82] | 76.7 (22.04) [68.81, 84.59] | 75.5 (18.96) [68.72, 82.28] | 73.9 (22.74) [65.76, 82.04] | 67.6 (28.32) [57.47, 77.73] | 66.97 (27.17) [57.24, 76.69] | 64.23 (31.77) [52.86, 75.6] |
|  |  | avoidance | 30.06 (27.13) [20.35, 39.77] | 12.7 (21.27) [5.09, 20.31] | 13.3 (17.46) [7.05, 19.55] | 18.07 (21.47) [10.38, 25.75] | 21.8 (23.95) [13.23, 30.37] | 29.93 (26.86) [20.32, 39.55] | 29.17 (29.99) [18.44, 39.9] | 39.6 (33.51) [27.61, 51.59] | 46.6 (35.95) [33.74, 59.46] | 59.4 (33.71) [47.34, 71.46] |
|  | Mood [4-20] | mood valence | 15.46 (3.26) [14.29, 16.63] | 16.17 (3.32) [14.98, 17.36] | - | 16.5 (2.86) [15.48, 17.52] | - | 16.27 (2.33) [15.43, 17.1] | - | 14.37 (3.97) [12.95, 15.79] | - | 14 (3.82) [12.63, 15.37] |
|  |  | calmness | 15.17 (3.21) [14.02, 16.31] | 15.57 (3.73) [14.23, 16.9] | - | 16.1 (2.28) [15.28, 16.92] | - | 15.47 (3.21) [14.32, 16.62] | - | 15.27 (2.75) [14.28, 16.25] | - | 13.43 (4.07) [11.98, 14.89] |
|  |  | energetic arousal | 13.39 (3.43) [12.17, 14.62] | 13.5 (3.76) [12.16, 14.84] | - | 14.53 (3.1) [13.42, 15.64] | - | 14.07 (3.07) [12.97, 15.17] | - | 12.8 (3.68) [11.48, 14.12] | - | 12.07 (3.54) [10.8, 13.33] |
|  | Fatigue [1-5] | general fatigue | 2.21 (1.09) [1.82, 2.61] | 1.97 (1.22) [1.53, 2.4] | - | 1.77 (1.04) [1.39, 2.14] | - | 1.97 (1) [1.61, 2.32] | - | 2.43 (1.1) [2.04, 2.83] | - | 2.93 (1.11) [2.54, 3.33] |
|  |  | physical fatigue | 3.03 (1.12) [2.63, 3.43] | 2.67 (1.09) [2.28, 3.06] | - | 2.8 (1.06) [2.42, 3.18] | - | 3.03 (0.93) [2.7, 3.37] | - | 3.33 (1.18) [2.91, 3.76] | - | 3.3 (1.32) [2.83, 3.77] |
|  |  | mental fatigue | 2.67 (1.04) [2.3, 3.05] | 2.33 (0.99) [1.98, 2.69] | - | 2.33 (0.96) [1.99, 2.68] | - | 2.63 (0.89) [2.31, 2.95] | - | 2.97 (1.22) [2.53, 3.4] | - | 3.1 (1.16) [2.69, 3.51] |
|  |  | reduced motivation | 2.3 (1.2) [1.87, 2.73] | 1.97 (1.1) [1.57, 2.36] | - | 2.03 (1.07) [1.65, 2.41] | - | 2.1 (0.96) [1.76, 2.44] | - | 2.77 (1.45) [2.25, 3.29] | - | 2.63 (1.4) [2.13, 3.13] |
|  |  | reduced activity | 3.25 (1.19) [2.83, 3.68] | 3.2 (1.06) [2.82, 3.58] | - | 3.07 (1.23) [2.63, 3.51] | - | 3.17 (1.15) [2.76, 3.58] | - | 3.43 (1.19) [3.01, 3.86] | - | 3.4 (1.33) [2.92, 3.88] |
|  | Physiolog-ical stress responses | sAA in U/mL | 60.73 (49.98) [42.85, 78.62] | 63.64 (60.82) [41.88, 85.4] | 71.6 (59.66) [50.26, 92.95] | 76.75 (47.72) [59.68, 93.83] | 61.6 (63.11) [39.02, 84.19] | 58.66 (42.58) [43.42, 73.89] | 46.67 (29.62) [36.07, 57.27] | 57.96 (45.41) [41.71, 74.21] | 54.43 (45.07) [38.3, 70.55] | 55.28 (55.82) [35.3, 75.25] |
|  |  | sCort in nmol/L | 2.45 (1.78) [1.81, 3.09] | 5 (2.83) [3.99, 6.01] | 3.26 (1.97) [2.55, 3.96] | 2.61 (1.52) [2.07, 3.15] | 2.05 (1.13) [1.64, 2.45] | 1.86 (1.21) [1.43, 2.29] | 1.69 (0.83) [1.4, 1.99] | 1.76 (0.97) [1.42, 2.11] | 1.95 (2.54) [1.04, 2.85] | 1.89 (3.04) [0.81, 2.98] |
|  |  | heart rate (bpm)^1^ | 73.18 (9.93) [72.69, 73.66] | - | 77.49 (10.16) [76.99, 77.99] | 75.05 (9.89) [74.56, 75.53] | 71.1 (9.5) [70.63, 71.56] | 72.12 (10.14) [71.62, 72.61] | 71.69 (9.83) [71.21, 72.17] | 72.75 (9.95) [72.26, 73.23] | 72.14 (9.39) [71.69, 72.6] | 73.09 (10.58) [72.58, 73.61] |
| ^1^ descriptive statistics for heart rate refer to a period including preceding hour (e.g. 12:00 is in fact 11:00 – 12:00) | | | | | | | | | | | | |

Table S3. Means, standard deviations, and confidence intervals of dependent variables for the morning after each session of the lab study

| Dependent variable | | Condition | | | | | |  |
| --- | --- | --- | --- | --- | --- | --- | --- | --- |
|  |  | Baseline | | | Isolation | | Fasting |  |
|  |  | *M* (*SD*) 95% *CI* | | | | | |  |
| Motivation  [1-100] | loneliness | | 13.62 (20.39) [6.33, 20.92] | 14.71 (17.67) [8.39, 21.04] | | 16.07 (20.88) [8.6, 23.54] | | |
|  | desire for soc. contact | | 34.55 (25.24) [25.52, 43.58] | 33.11 (28.18) [23.02, 43.19] | | 34.54 (24.29) [25.84, 43.23] | | |
|  | hunger | | 37.1 (28.28) [26.98, 47.22] | 44.43 (29.53) [33.86, 55] | | 42.29 (29.18) [31.84, 52.73] | | |
|  | desire for food | | 38.9 (28.61) [28.66, 49.13] | 43.29 (29.52) [32.72, 53.85] | | 42.39 (30.8) [31.37, 53.41] | | |
| Stress [1-100] | stress | | 2.43 (4.12) [0.95, 3.9] | 7.14 (15.33) [1.66, 12.63] | | 6.7 (16.9) [0.65, 12.75] | | |
|  | anxiety | | 2.64 (6.4) [0.35, 4.93] | 1.43 (4.25) [-0.09, 2.95] | | 2.63 (6.31) [0.38, 4.89] | | |
|  | controllability | | 21.54 (38.32) [7.82, 35.25] | 15.14 (30.76) [4.14, 26.15] | | 17.67 (35.5) [4.96, 30.37] | | |
|  | avoidance | | 3.32 (1.83) [2.67, 3.98] | 5.71 (14.85) [0.4, 11.03] | | 6.83 (17.9) [0.43, 13.24] | | |
| Mood [4-20] | mood valence | | 15.21 (2.61) [14.27, 16.14] | 14.59 (2.8) [13.59, 15.59] | | 14.17 (2.98) [13.1, 15.23] | | |
|  | calmness | | 16.86 (2.22) [16.07, 17.65] | 16.38 (3.2) [15.23, 17.52] | | 16.23 (2.97) [15.17, 17.3] | | |
|  | energetic arousal | | 14.21 (3.66) [12.9, 15.52] | 13.38 (3.84) [12.01, 14.75] | | 14 (3.47) [12.76, 15.24] | | |
| Fatigue [1-5] | general fatigue | | 1.55 (0.91) [1.23, 1.88] | 1.9 (1.11) [1.5, 2.29] | | 1.83 (1.09) [1.44, 2.22] | | |
|  | physical fatigue | | 2.66 (1.26) [2.2, 3.11] | 2.86 (1.19) [2.44, 3.29] | | 2.6 (1.04) [2.23, 2.97] | | |
|  | mental fatigue | | 2.34 (1.11) [1.95, 2.74] | 2.61 (0.96) [2.27, 2.95] | | 2.37 (1) [2.01, 2.72] | | |
|  | reduced motivation | | 1.93 (1.13) [1.53, 2.34] | 2.07 (1.1) [1.68, 2.46] | | 1.73 (1.05) [1.36, 2.11] | | |
|  | reduced activity | | 2.62 (1.15) [2.21, 3.03] | 3 (1.13) [2.59, 3.41] | | 2.82 (1.16) [2.41, 3.24] | | |
| Physiological stress responses | sAA S1 in U/mL | | 34.65 (28.91) [24.31, 45] | 41.48 (35.7) [28.71, 54.26] | | 36.83 (33.85) [24.72, 48.94] | | |
|  | AAR in U/mL | | -8.01 (37.39) [-21.4, 5.37] | -13.07 (28.41) [-23.24, -2.9] | | -5.71 (38.2) [-19.37, 7.96] | | |
|  | sCort S1 in nmol/L | | 6.58 (3.74) [5.24, 7.92] | 6.81 (3.97) [5.39, 8.23] | | 5.96 (3.11) [4.85, 7.08] | | |
|  | CAR in nmol/L | | 3.04 (3.61) [1.75, 4.33] | 2.19 (4.7) [0.51, 3.87] | | 3.17 (3.8) [1.81, 4.52] | | |

Table S4. Overview of the results from the experimental and the field study.

Model estimates are expressed in standard deviations of the respective dependent measure for the sake of comparability of effect sizes.

| Dependent variable |  | Lab study | | | |  | Field study | | | | | |
| --- | --- | --- | --- | --- | --- | --- | --- | --- | --- | --- | --- | --- |
|  |  | Social isolation^1^ | Social isolation * time | Food deprivation^1^ | Food deprivation * time |  | Social isolation^1^ | Social isolation * time | Social isolation * Sociability (SRQ) | Social isolation * Living alone | Social isolation * Chronic stress (PSS) | |
|  |  | β (*SE*), *p* value | | | |  | β (*SE*), *p* value | | | | | |
| Motivation (VAS) | loneliness | 0.51 (0.13) **.001** | 0.07 (0.02), **.002** | 0.10 (0.12), .400 | 4*10^-3^ (0.02), .838 |  | 0.12 (0.04), **.007** | 0.02 (0.01), 0.181 | -0.03 (0.03), .288 | -0.18 (0.09), .054 | 0.01 (0.01), .107 | |
|  | desire for soc. contact | 0.49 (0.13), **.001** | 0.08 (0.02), **< .001** | 0.14 (0.12), .250 | 0.01 (0.02), .659 |  | -0.11 (0.05), **.031** | 0.02 (0.01), .190 | -0.07(0.03), .034 | 0.09 (0.11), .411 | -0.01 (0.01), .374 | |
|  | hunger | 0.03 (0.08), .725 | -1*10^-3^ (0.02), .967 | 1.04 (0.08), **< .001** | 0.29 (0.02), **< .001** |  | - | - | - | - | - | |
|  | desire for food | 0.07 (0.08), .382 | -0.01 (0.02), .572 | 1.01 (0.08), **< .001** | 0.26 (0.02), **< .001** |  | - | - | - | - | - | |
| Stress  (VAS; von Dawans et al., 2011) | stress | -0.01 (0.1), .919 | 0.02 (0.02), .339 | 0.11 (0.11), .301 | 0.01 (0.03), .740 |  | 0.02 (0.05), .791 | 0.02 (0.01), .141 | -0.02 (0.04), .570 | 0.01 (0.13), .959 | 0.01 (0.01), .283 | |
|  | anxiety | 0.08 (0.07), .273 | 0.02 (0.02), .409 | 0.05 (0.13), .686 | 4*10^-3^ (0.02), .888 |  | - | - | - | - | - | |
|  | control. | -0.11 (0.09), .203 | -0.01 (0.02), .726 | -0.28 (0.11), **.015** | -0.06 (0.03), **.048** |  | - | - | - | - | - | |
|  | avoidance | 0.31 (0.11), **.008** | 0.02 (0.02), .125 | 0.35 (0.10), **.001** | 0.06 (0.02), **< .001** |  | - | - | - | - | - | |
| Mood  (MDBF; Steyer et al., 2003) | mood valence | -0.19 (0.16), .237 | -0.03 (0.02), .209 | -0.45 (0.12), **< .001** | -0.07 (0.02), **.002** |  | -0.10 (0.05), .069 | -0.01 (0.01), .306 | -0.02 (0.04), .511 | 0.10 (0.12), .423 | -0.01 (0.01), .316 | |
|  | calmness | 0.03 (0.15), .855 | -0.03 (0.03), .082 | -0.27 (0.13), **.048** | -0.05 (0.03), **.044** |  | 0.04 (0.06), .475 | -0.02(0.01), .078 | -0.03 (0.04), .435 | 0.07, 0.12, .554 | -0.01 (0.01), .391 | |
|  | energetic arousal | -0.48 (0.12), **< .001** | -0.01 (0.03), .644 | -0.36 (0.11), **.003** | -0.01 (0.03), .723 |  | -0.09 (0.06), .146 | 0.01 (0.01), .594 | -0.08 (0.04), **.036** | 0.31 (0.13), **.019** | 0.01 (0.01), .101 | |
| Fatigue  (MFI; Lin et al., 2009; Doerr et al., 2021) | general fatigue | 0.34 (0.11), **.004** | 0.04 (0.03), .203 | 0.40 (0.11), **.001** | 0.07 (0.03), **.017** |  | 0.08 (0.05), .207 | 3*10^-4^(0.01), .978 | 0.06 (0.04), .164 | -0.09 (0.13), .509 | -2*10^-3^ (0.01), .799 | |
|  | physical fatigue | 0.21 (0.12), .094 | 0.03 (0.02), .138 | 0.24 (0.13), .062 | 0.03 (0.02), .157 |  | - | - | - | - |  | |
|  | mental fatigue | 0.17 (0.11), .137 | 0.01 (0.03), .750 | 0.26 (0.10), **.012** | 0.01 (0.03), .832 |  | - | - | - | - | - | |
|  | reduced motivation | 0.23 (0.11), **.046** | 0.04 (0.02), .068 | 0.38 (0.10), **.001** | 0.04 (0.02), .078 |  | - | - | - | - | - | |
|  | reduced activity | 0.33 (0.12), **.011** | 0.04 (0.03), .182 | 0.30 (0.12), **.015** | 0.01 (0.03), .743 |  | - | - | - | - | - | |
| Physiological stress responses (moment. values, AUCg, AUCi)^2^ | sCort m.v.^3^ | 0.06 (0.06), .928 | 0.04 (0.02), .116 | -0.14 (0.08), .227 | 0.03 (0.02), .290 |  | - | - | - | - | - | |
|  | sCort AUCg | -0.02 (0.13), 1 | - | -0.27 (0.13), .126 | - |  | - | - | - | - | - | |
|  | sCort AUCi | 0.08 (0.15), 1 | - | 0.04 (0.15), 1 | - |  | - | - | - | - | - | |
|  | sAA m.v.^3^ | 0.01 (0.07), 1 | -0.01 (0.02), 1 | 0.04 (0.09), 1 | -0.07 (0.02), **.001** |  | - | - | - | - | - | |
|  | sAA AUCg | -0.04 (0.09), 1 | - | 0.03 (0.09), 1 | - |  | - | - | - | - | - | |
|  | sAA AUCi | 0.18 (0.25), 1 | - | -0.04 (0.25), 1 | - |  | - | - | - | - | - | |
|  | heart rate m.v.^3^ | 0.04 (0.1), 1 | 2*10^-3^ (0.02), 1 | -0.25 (0.06), **.001** | -0.05 (0.02), **.003** |  | - | - | - | - | - | |
|  | heart rate AUCg | 0.06 (0.11), 1 | - | -0.35 (0.12), **.008** | - |  | - | - | - | - | - | |
|  | heart rate AUCi | -0.08 (0.20), 1 | - | -0.07 (0.20), 1 | - |  | - | - | - | - | - | |
| ^1^compared to baseline condition/day, ^2^ all p values are Bonferroni corrected for multiple comparisons: momentary values, area under curve with respect to ground (AUCg), and area under curve with respect to increase (AUCi), ^3^ momentary values | | | | | | | | | | | |  |

Table S5. Results of momentary salivary cortisol and alpha amylase on the full sample before outlier exclusion, with raw data

| Dependent variable | | Social isolation^1^ | Social isolation * time | Food deprivation^1^ | Food deprivation * time |
| --- | --- | --- | --- | --- | --- |
|  |  | β (*SE*), *p* value | | | |
| Physiological stress responses (moment. values)^2^ | sCort | -0.02 (0.12), 1 | 0.06 (0.04), .603 | -0.19 (0.12), .298 | 0.09 (0.05), .149 |
|  | sAA | -2.78 (3.41), 1 | 0.58 (0.93), 1 | 0.94 (3.91), 1 | -2.66 (0.93), **.013** |
| ^1^compared to baseline condition, ^2^all p values are Bonferroni corrected for multiple comparisons: momentary values, area under curve with respect to ground (AUCg), and area under curve with respect to increase (AUCi) | | | | | |

Table S6. Results of momentary salivary cortisol and alpha amylase on the full sample before outlier exclusion, with transformed data (log10).

| Dependent variable | | Social isolation^1^ | Social isolation * time | Food deprivation^1^ | Food deprivation * time |
| --- | --- | --- | --- | --- | --- |
|  |  | β (*SE*), *p* value | | | |
| Physiological stress responses (moment. values)^2^ | sCort | 0.01 (0.02), 1 | 0.01 (0.01), .075 | -0.05 (0.03), .123 | 0.01 (0.01), .524 |
|  | sAA | -2*10^-3^ (0.02), 1 | -2*10^-3^ (0.01), 1 | 0.01 (0.03), 1 | - 0.02 (0.01), **.002** |
| ^1^compared to baseline condition, ^2^all p values are Bonferroni corrected for multiple comparisons: momentary values, area under curve with respect to ground (AUCg), and area under curve with respect to increase (AUCi) | | | | | |

Table S7. Overview of the results from the morning after each experimental session.

| Dependent variable | | Social isolation^1^ | Food deprivation^1^ |
| --- | --- | --- | --- |
|  |  | β (*SE*), *p* value | |
| Motivation (VAS) | loneliness | 1.22 (4.39), .781 | 3.07 (4.44), .491 |
|  | desire for soc. contact | -4.31 (6.05), .479 | -0.06 (6.12), .992 |
|  | hunger | 4.73 (4.26), .272 | 5.11 (4.32), .242 |
|  | desire for food | 1.52 (4.65), .745 | 3.27 (4.71), .490 |
| Stress  (VAS; von Dawans et al., 2011) | stress | 4.96 (2.98), .102 | 4.70 (2.94), .116 |
|  | anxiety | -1.11 (0.71), .124 | -0.07 (0.70), .927 |
|  | controllability | -3.34 (1.87), .079 | -2.73 (1.84), .144 |
|  | avoidance | 2.64 (2.83), .355 | 3.82 (2.79), .178 |
| Mood  (MDBF; Steyer et al., 2003) | mood valence | -0.64 (0.66), .334 | -0.89 (0.66), .181 |
|  | calmness | -0.50 (0.60), 411 | -0.36 (0.60), .556 |
|  | energetic arousal | -0.68 (0.89), .449 | -0.25 (0.89), .780 |
| Fatigue  (MFI; Lin et al., 2009; Doerr et al., 2021) | general fatigue | 0.36 (0.25), .163 | 0.21 (0.25), .400 |
|  | physical fatigue | 0.07 (0.25), .781 | -0.14 (0.26), .578 |
|  | mental fatigue | 0.18 (0.23), .435 | 4*10^-15^ (0.23), 1 |
|  | reduced motivation | 0.14 (0.22), .511 | -0.18 (0.22), .412 |
|  | reduced activity | 0.36 (0.22), .111 | 0.15 (0.23), .508 |
| Physiological stress response (waking level: S1, awakening responses: CAR and AAR)^2^ | sCort S1 in nmol/L | 0.01 (0.06), .825 | -0.05, (0.06), .353 |
|  | CAR in nmol/L | -0.82 (0.98), .406 | -0.11 (0.98), .912 |
|  | sAA S1 in U/mL | 0.07 (0.07), .331 | 0.01 (0.07), .921 |
|  | AAR in U/mL | -5.89 (9.64), .543 | 1.17 (9.64), .904 |
| ^1^compared to baseline condition, ^2^all p values are Bonferroni corrected for multiple comparisons: momentary values, area under curve with respect to ground (AUCg), and area under curve with respect to increase (AUCi) | | | |

# Field study

S8. Comparison of the field sample to population norms

The average UCLA Loneliness score was 41.57 (SD = 9.87), close to the normative mean for students (M= 40.08, SD= 9.5) and higher than the normative mean for elderly (M= 31.5, SD= 6.9). Chronic stress as measured by Perceived Stress Scale (PSS) (Klein et al., 2016; Mondo et al., 2021) was 19.5 (SD = 7.48), higher than the score found on an Austrian representative sample during the same period (M = 15.97, SD = 7.47) (Pieh et al., 2020).

S9. General information about linear mixed models in the field study

All models included participant as a clustering variable and the structure of random effects was kept “maximal” (Barr et al., 2013) whereby there were random slopes and intercepts for all main effects and interactions where possible. Variables type of day and free time were participant mean centered (Enders & Tofighi, 2007). The continuous person-related predictors (sociability and chronic stress) were grand mean centered.

Table S8. Between-person means, standard deviations, and confidence intervals of dependent variables for the field study

| Type of day | Dependent variable | | |  | | Measurement point | | | | | | | |
| --- | --- | --- | --- | --- | --- | --- | --- | --- | --- | --- | --- | --- | --- |
|  |  |  |  | Overall | | 10:00 – 11:00 | | 11:00 – 14:00 | | 14:00 – 17:00 | | 17:00 – 20:00 | |
|  |  |  |  | *M* (between-person *SD*) 95% *CI* | | | | | | | | | |
| Baseline | Motivation [1-100] | loneliness | 33.47 (27.68) [27.65, 39.29] | | 33.43 (28.44) [27.46, 39.41] | | 32.96 (26.14) [27.46, 38.45] | | 33.49 (27.31) [27.75, 39.23] | | 34 (28.83) [27.94, 40.06] | |  |
|  |  | desire for soc. contact | 40.93 (25.84) [35.5, 46.36] | | 42.64 (25.52) [37.28, 48.01] | | 39.85 (25.9) [34.4, 45.29] | | 40.36 (25.69) [34.96, 45.76] | | 40.86 (26.24) [35.34, 46.37] | |  |
|  | Stress [1-100] | stress | 32.57 (21.03) [28.15, 36.99] | | 35.88 (21.43) [31.38, 40.38] | | 32.94 (21.27) [28.48, 37.41] | | 30.49 (19.09) [26.48, 34.5] | | 30.98 (22.34) [26.28, 35.67] | |  |
|  | Mood [1-100] | mood valence | 62.44 (17.79) [58.71, 66.18] | | 61.19 (18.02) [57.4, 64.97] | | 63.17 (17.92) [59.41, 66.94] | | 62.21 (16.05) [58.84, 65.58] | | 63.21 (19.19) [59.18, 67.24] | |  |
|  |  | calmness | 60.73 (17.64) [57.02, 64.43] | | 58.65 (16.86) [55.11, 62.2] | | 61.79 (18.22) [57.96, 65.62] | | 61.28 (16.15) [57.89, 64.67] | | 61.18 (19.33) [57.12, 65.24] | |  |
|  |  | energetic arousal | 57.3 (16.86) [53.76, 60.84] | | 56.48 (19.48) [52.39, 60.57] | | 59.96 (16.63) [56.47, 63.46] | | 58.47 (14.66) [55.39, 61.55] | | 54.29 (16.68) [50.79, 57.8] | |  |
|  | Fatigue [1-100] | general fatigue | 35.49 (21.03) [31.07, 39.91] | | 33.16 (22.7) [28.39, 37.93] | | 32.92 (20.17) [28.69, 37.16] | | 35.9 (20.17) [31.66, 40.14] | | 39.99 (21.09) [35.56, 44.42] | |  |
| Isolation | Motivation [1-100] | loneliness | 36.72 (30.87) [30.23, 43.21] | | 35.4 (29.8) [29.14, 41.66] | | 34.78 (31.53) [28.15, 41.4] | | 37.94 (30.49) [31.53, 44.34] | | 38.78 (31.68) [32.12, 45.44] | |  |
|  |  | desire for soc. contact | 36.98 (28.69) [30.95, 43.01] | | 36.73 (27.98) [30.85, 42.61] | | 34.36 (28.76) [28.32, 40.4] | | 38.5 (29.49) [32.3, 44.7] | | 38.35 (28.53) [32.35, 44.34] | |  |
|  | Stress [1-100] | stress | 33.01 (24.42) [27.88, 38.14] | | 33.21 (22.2) [28.54, 37.87] | | 34.12 (24.14) [29.05, 39.19] | | 31.22 (24.69) [26.03, 36.4] | | 33.5 (26.65) [27.9, 39.09] | |  |
|  | Mood [1-100] | mood valence | 60.44 (20.42) [56.15, 64.73] | | 60.08 (20.9) [55.68, 64.47] | | 61.37 (19.23) [57.33, 65.41] | | 60.92 (20.74) [56.56, 65.28] | | 59.4 (20.79) [55.03, 63.76] | |  |
|  |  | calmness | 59.86 (20.61) [55.52, 64.19] | | 60.09 (20.54) [55.77, 64.4] | | 61.63 (20.03) [57.42, 65.84] | | 59.46 (20.95) [55.06, 63.86] | | 58.25 (20.93) [53.85, 62.65] | |  |
|  |  | energetic arousal | 55.03 (19.99) [50.82, 59.23] | | 54.56 (22.95) [49.74, 59.39] | | 58.87 (19.33) [54.81, 62.93] | | 52.93 (18.68) [49, 56.85] | | 53.74 (19.02) [49.75, 57.74] | |  |
|  | Fatigue [1-100] | general fatigue | 37.49 (23.99) [32.45, 42.53] | | 32.7 (24.25) [27.61, 37.8] | | 33.49 (22.19) [28.83, 38.16] | | 43.82 (24.71) [38.62, 49.01] | | 39.95 (24.81) [34.74, 45.16] | |  |

Table S9. Post-hoc analyses – effects of gender in the field study

| Dependent variable | | Gender^1^ | Social isolation * gender |
| --- | --- | --- | --- |
|  |  | β (*SE*), *p* value | |
| Motivation (VAS) | loneliness | 3.66 (5.40), .499 | -4.28 (2.65), .110 |
|  | desire for soc. contact | 3.21 (4.89), .514 | 4.45 (2.88), .127 |
| Stress (VAS) | stress | 2.05 (3.95), .606 | -1.98 (2.89), .495 |
| Mood  (MDBF; Wilhelm & Schoebi, 2007) | mood valence | -2.81 (3.78), .408 | 3.12 (2.38), .194 |
|  | calmness | -1.51 (3.29), .648 | 3.49 (2.36), .142 |
|  | energetic arousal | 2.95 (2.68), .275 | 3.49 (2.36), .142 |
| Fatigue  (VAS) | general fatigue | -2.76 (3.63), .450 | -4.77 (3.10), .128 |
| ^1^female gender is the reference variable level (0) | | | |

# References

Barr, D. J., Levy, R., Scheepers, C., & Tily, H. J. (2013). Random effects structure for confirmatory hypothesis testing: Keep it maximal. *Journal of Memory and Language*, *68*(3). https://doi.org/10.1016/j.jml.2012.11.001

Buysse, D. J., Reynolds, C. F., Monk, T. H., Berman, S. R., & Kupfer, D. J. (1989). The Pittsburgh Sleep Quality Index: A new instrument for psychiatric practice and research. *Psychiatry Research*, *28*(2), 193–213. https://doi.org/10.1016/0165-1781(89)90047-4

Cacioppo, J. T., Cacioppo, S., & Boomsma, D. I. (2014). Evolutionary Mechanisms for Loneliness. *Cognition & Emotion*, *28*(1). https://doi.org/10.1080/02699931.2013.837379

Dalbert, C. (1992). Subjektives Wohlbefinden junger Erwachsener: Theoretische und empirische Analysen der Struktur und Stabilität. [Young adults’ subjective well-being: Theoretical and empirical analyses of its structure and stability.]. *Zeitschrift Für Differentielle Und Diagnostische Psychologie*, *13*(4), 207–220.

Doerr, J. M., Nater, U. M., Feneberg, A. C., & Mewes, R. (2021). Differential associations between fatigue and psychobiological stress measures in women with depression and women with somatic symptom disorder. *Psychoneuroendocrinology*, *132*, 105343. https://doi.org/10.1016/j.psyneuen.2021.105343

Enders, C. K., & Tofighi, D. (2007). Centering predictor variables in cross-sectional multilevel models: A new look at an old issue. *Psychological Methods*, *12*(2), 121–138. https://doi.org/10.1037/1082-989X.12.2.121

Grant, N., Hamer, M., & Steptoe, A. (2009). Social Isolation and Stress-related Cardiovascular, Lipid, and Cortisol Responses. *Annals of Behavioral Medicine*, *37*(1), 29–37. https://doi.org/10.1007/s12160-009-9081-z

Hilbert, A., Tuschen-Caffier, B., Karwautz, A., Niederhofer, H., & Munsch, S. (2007). Eating Disorder Examination-Questionnaire. *Diagnostica*, *53*(3), 144–154. https://doi.org/10.1026/0012-1924.53.3.144

Hughes, J. (2021). reghelper: Helper Functions for Regression Analysis. *R Package Version 1.0.2*.

Klaus, K., Doerr, J. M., Strahler, J., Skoluda, N., Linnemann, A., & Nater, U. M. (2019). Poor night’s sleep predicts following day’s salivary alpha-amylase under high but not low stress. *Psychoneuroendocrinology*, *101*, 80–86. https://doi.org/10.1016/j.psyneuen.2018.10.030

Klein, E. M., Brähler, E., Dreier, M., Reinecke, L., Müller, K. W., Schmutzer, G., Wölfling, K., & Beutel, M. E. (2016). The German version of the Perceived Stress Scale–psychometric characteristics in a representative German community sample. *BMC Psychiatry*, *16*(1), 1–10.

Kuznetsova, A., Brockhoff, P. B., & Christensen, R. H. B. (2017). lmerTest Package: Tests in Linear Mixed Effects Models. *Journal of Statistical Software*, *82*(1), Article 1. https://doi.org/10.18637/jss.v082.i13

Lin, J.-M. S., Brimmer, D. J., Maloney, E. M., Nyarko, E., BeLue, R., & Reeves, W. C. (2009). Further validation of the Multidimensional Fatigue Inventory in a US adult population sample. *Population Health Metrics*, *7*(1), 18. https://doi.org/10.1186/1478-7954-7-18

Linnemann, A., Strahler, J., & Nater, U. M. (2017). Assessing the Effects of Music Listening on Psychobiological Stress in Daily Life. *Journal of Visualized Experiments*, *120*. https://doi.org/10.3791/54920

Long, J. A. (2019). interactions: Comprehensive, User-Friendly Toolkit for Probing Interactions. *R Package Version 1.1.0*. https://cran.r-project.org/package=interactions

Maner, J. K., & Miller, S. L. (2014). Hormones and social monitoring: Menstrual cycle shifts in progesterone underlie women’s sensitivity to social information. *Evolution and Human Behavior*, *35*(1), 9–16. https://doi.org/10.1016/j.evolhumbehav.2013.09.001

Mondo, M., Sechi, C., & Cabras, C. (2021). Psychometric evaluation of three versions of the Italian perceived stress scale. *Current Psychology*, *40*(4), 1884–1892.

Pieh, C., Budimir, S., & Probst, T. (2020). The effect of age, gender, income, work, and physical activity on mental health during coronavirus disease (COVID-19) lockdown in Austria. *Journal of Psychosomatic Research*, *136*, 110186. https://doi.org/10.1016/j.jpsychores.2020.110186

Powell, M. (2009). The BOBYQA Algorithm for Bound Constrained Optimization without Derivatives. *Technical Report, Department of Applied Mathematics and Theoretical Physics*.

Pruessner, J. C., Kirschbaum, C., Meinlschmid, G., & Hellhammer, D. H. (2003). Two formulas for computation of the area under the curve represent measures of total hormone concentration versus time-dependent change. *Psychoneuroendocrinology*, *28*(7), 916–931. https://doi.org/10.1016/S0306-4530(02)00108-7

Quoidbach, J., Taquet, M., Desseilles, M., de Montjoye, Y.-A., & Gross, J. J. (2019). Happiness and Social Behavior. *Psychological Science*, *30*(8), 1111–1122. https://doi.org/10.1177/0956797619849666

Rø, Ø., Reas, D. L., & Stedal, K. (2015). Eating Disorder Examination Questionnaire (EDE-Q) in Norwegian Adults: Discrimination between Female Controls and Eating Disorder Patients: Global EDE-Q Cut-Off. *European Eating Disorders Review*, *23*(5), 408–412. https://doi.org/10.1002/erv.2372

Russell, D. W. (1996). UCLA Loneliness Scale (Version 3): Reliability, Validity, and Factor Structure. *Journal of Personality Assessment*, *66*(1), 20–40. https://doi.org/10.1207/s15327752jpa6601_2

Schulz, P., Schlotz, W., & Becker, P. (2004). *Trierer Inventar zum Chronischen Stress (TICS) [Trier Inventory for Chronic Stress (TICS)]* [Monograph]. https://eprints.soton.ac.uk/50017/

Stalder, T., Kirschbaum, C., Kudielka, B. M., Adam, E. K., Pruessner, J. C., Wüst, S., Dockray, S., Smyth, N., Evans, P., Hellhammer, D. H., Miller, R., Wetherell, M. A., Lupien, S. J., & Clow, A. (2016). Assessment of the cortisol awakening response: Expert consensus guidelines. *Psychoneuroendocrinology*, *63*, 414–432. https://doi.org/10.1016/j.psyneuen.2015.10.010

Steyer, R., Schwenkmezger, P., Notz, P., & Eid, M. (2003). *Development of the Multidimensional Mood State Questionnaire (MDBF). Primary dataEntwicklung des Mehrdimensionalen Befindlichkeitsfragebogens (MDBF). Primärdatensatz* [Data set]. ZPID Leibniz Institute for Psychology Information. https://doi.org/10.5160/psychdata.srrf91en15

Strahler, J., Skoluda, N., Kappert, M. B., & Nater, U. M. (2017). Simultaneous measurement of salivary cortisol and alpha-amylase: Application and recommendations. *Neuroscience & Biobehavioral Reviews*, *83*, 657–677. https://doi.org/10.1016/j.neubiorev.2017.08.015

Tingley, D., Yamamoto, T., Hirose, K., Keele, L., & Imai, K. (2014). **mediation**: *R* Package for Causal Mediation Analysis. *Journal of Statistical Software*, *59*(5). https://doi.org/10.18637/jss.v059.i05

van Doorn, J., van den Bergh, D., Böhm, U., Dablander, F., Derks, K., Draws, T., Etz, A., Evans, N. J., Gronau, Q. F., Haaf, J. M., Hinne, M., Kucharský, Š., Ly, A., Marsman, M., Matzke, D., Gupta, A. R. K. N., Sarafoglou, A., Stefan, A., Voelkel, J. G., & Wagenmakers, E.-J. (2021). The JASP guidelines for conducting and reporting a Bayesian analysis. *Psychonomic Bulletin & Review*, *28*(3), 813–826. https://doi.org/10.3758/s13423-020-01798-5

von Dawans, B., Kirschbaum, C., & Heinrichs, M. (2011). The Trier Social Stress Test for Groups (TSST-G): A new research tool for controlled simultaneous social stress exposure in a group format. *Psychoneuroendocrinology*, *36*(4), 514–522. https://doi.org/10.1016/j.psyneuen.2010.08.004
